# Antimicrobial Potential of Polyphenols: Mechanisms of Action and Microbial Responses—A Narrative Review

**DOI:** 10.3390/antiox14020200

**Published:** 2025-02-10

**Authors:** Luca De Rossi, Gabriele Rocchetti, Luigi Lucini, Annalisa Rebecchi

**Affiliations:** 1 Department for Sustainable Food Process (DiSTAS), Università Cattolica del Sacro Cuore, Via Stefano Leonida Bissolati 74, 26100 Cremona, Italy; luca.derossi@unicatt.it (L.D.R.); annalisa.rebecchi@unicatt.it (A.R.); 2 Department of Animal Science, Food and Nutrition, Università Cattolica del Sacro Cuore, Via Emilia Parmense 84, 29122 Piacenza, Italy; 3 Department for Sustainable Food Process, Università Cattolica del Sacro Cuore, Via Emilia Parmense 84, 29122 Piacenza, Italy; luigi.lucini@unicatt.it

**Keywords:** polyphenols, antimicrobial activity, foodborne pathogens, antioxidants, plant extracts

## Abstract

Polyphenols (PPs) are recognized as bioactive compounds and antimicrobial agents, playing a critical role in enhancing food safety, preservation, and extending shelf life. The antimicrobial effectiveness of PPs has different molecular and biological reasons, predominantly linked to their hydroxyl groups and electron delocalization, which interact with microbial cell membranes, proteins, and organelles. These interactions may reduce the efficiency of metabolic pathways, cause destructive damage to the cell membrane, or they may harm the proteins and nucleic acids of the foodborne bacteria. Moreover, PPs exhibit a distinctive ability to form complexes with metal ions, further amplifying their antimicrobial activity. This narrative review explores the complex and multifaceted interactions between PPs and foodborne pathogens, underlying the correlation of their chemical structures and mechanisms of action. Such insights shed light on the potential of PPs as innovative natural preservatives within food systems, presenting an eco-friendly and sustainable alternative to synthetic additives.

## 1. Introduction

**Figure 1:**
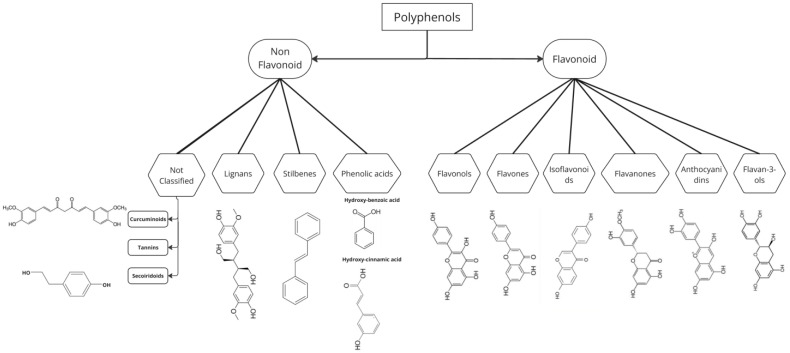
Classes, sub-classes, and generic molecular structure of polyphenols. The different chemical structures were represented using the MolView application (version 2.4).

Polyphenols (PPs) are secondary plant metabolites, primarily divided into two main categories: flavonoids and non-flavonoids. These categories are further subdivided into various subclasses based on the structure and number of substituent groups on the phenol units and linkage types [1]. Structurally, the flavonoid family is composed of two aromatic rings, C6(A)-C6(B), and this benzopyran skeleton is bridged by oxygen-cyclized C3(C) carbon to a tetrahydropyran ring [2,3]. As previously reported in the scientific literature [4,5,6], there are 43 distinct substructures of PPs grouped into five main classes: (i) flavonoids, (ii) lignans, (iii) phenolic acids, (iv) stilbenes, and (v) other PPs (Figure 1). Clearly, the classification of PPs is continually evolving due to their widespread distribution and the subtle variations in their molecular structures [6,7]. In general, PPs can be found in peels, husks, seeds, and leaves and exhibit a vast distribution of secondary compounds, estimated to be around 450,000 in number, with approximately 200,000 molecules identified and classified, serving various functions, including defence against biotic and abiotic stresses, in both plants and animals [8]. Additionally, considering their human and biological therapeutic potential, their application in food preservation is particularly compelling. Lacroix et al. [4] performed a functional pathway analysis of polyphenol-binding proteins, showing that multiple biochemical processes are influenced by PPs, including central metabolism and signaling pathways. Some proteins that interact with PPs often play a crucial role in detoxification processes and possess antioxidant properties, protecting cell structures from free radicals and cytotoxicity [9]. These beneficial properties have increased the interest in PPs. Moreover, their nutritional benefits, pharmacokinetic behaviours, therapeutic mechanisms, and potential health-promoting effects have made them one of the most studied compounds in nature [10]. PP interactions depend not only on the chemical classes but also on their ability to maintain a stable bond and, consequently, their bioavailability. Based on these interactions, PPs can be divided into two main categories: (i) free flavonoids (FFs), encompassing both free-flavonoid aglycones and free-phenolic acids, which may also occur in conjugated forms bound to low-molecular-weight compounds such as sugars, thus making them more available; and (ii) insoluble-bound phenolics (IBPs), which are either soluble (non-covalently bound to the matrix) or insoluble (covalently bound to the substrate). These are covalently linked to complex molecules, rendering them inaccessible until they reach the large intestine, where the microbiota can ferment and metabolize the residual structures [11].

The bioavailability of PPs, driven by their native structure and binding conditions, is also influenced by processes such as gastro-intestinal digestion, absorption, and metabolism, in addition to factors such as concentration in food, release in the food matrix, chemical structure, conjugation with other compounds, molecular size, degree of polymerization, and solubility [12]. Some parameters, such as molecular weight, degree of acylation or glycosylation, and conjugation with other phenolics, have been reported as allowing PPs to move through the double phospholipid layer, enabling different PPs to act in intracellular signaling processes and enhance key cellular behaviour, such as apoptosis, cell division, and overall growth [13]. One widely studied aspect that is related to all the previously mentioned interactions of PPs is their demonstrated antimicrobial activity against different foodborne Gram-positive bacteria (*Clostridium botulinum, Listeria monocytogenes, Staphylococcus aureus,* and *Clostridium perfringens*) and Gram-negative pathogens (*Salmonella* spp., *Campylobacter* spp., *Vibrio* spp. and *Escherichia coli*), as well as yeast, fungi, and moulds [1,3]. By studying these bacterial interactions, the European Food Safety Authority (EFSA) is starting to analyse more seriously the presence of PPs in our lives to help address the antimicrobial resistance (AMR) crisis by overcoming different bacterial resistances, thus containing the development of outbreaks [13,14,15]. Moreover, the well-established antioxidant activity of plant-derived PPs strongly supports the shift towards replacing chemical additives commonly used in the food industry [16,17]. At the same time, consumers are increasingly attracted by clean and green branded products due to health concerns related to synthetic additives. Thus, they want to apply sustainable practices and prefer minimally processed foods [7]. It is also important to mention that PPs are significantly influenced, both quantitively and qualitative, by genetics, the agroclimatic environment, exogenous conditions, harvesting time, and post-harvesting conditions, so the profiles of these compounds can change considerably [18]. Therefore, addressing current concerns regarding the concentration and dosage limits of PPs is imperative, as their effects in clinical trials often remain unclear due to significant interindividual variability [1].

The bioavailability of PPs is closely linked to their bioaccessibility. In particular, most PPs exhibit low solubility and cannot cross the lipid barrier or exist as glycosylated polymers, requiring enzymatic hydrolysis for absorption [19]. Strategies to enhance PP bioavailability, including nutritional interventions and technological/biotechnological approaches like microbial conversion into bioavailable metabolites, are already utilized [20]. For example, through different processing conditions (thus, methodology), it is possible to have a loss or improvement of phenolic accessibility ranging between -15% and +184% of p-Coumaric acid, ferulic acid, and sinapic acid in mechanically processed wheat bread. [21,22] Ribas–Agustì et al. 2018 [21] have also demonstrated that in enzymatically or chemically processed foods, it is possible to increase the bioavailability in a wide range, between 79% and 167% for vanillic acid and ferulic acid, respectively, processing whole wheat bread. In the same study, it has been proven that the developed extraction system can also enhance the ferulic acid bioaccessibility of 4-folds. The last comparison made on non-thermically processed food with different methodologies involved (such as high-pressure treatment, electric shocks, and sonication) has shown that the sonication, in specific conditions, can increase the total polyphenol concentration (TPC) to a peak of 400% for 2 min of treatment, decreasing at 300% at 10 min [23]. In vitro digestion models estimate the bioaccessibility of compounds, while in vivo studies assess bioavailability by measuring metabolites in blood and urine. However, bridging the gap between in vitro findings and in vivo outcomes remains a significant challenge. Therefore, current research focuses on optimizing methods to enhance the bioavailability and bioaccessibility of polyphenols (PPs) and evaluating their effects in human studies [20,22].

## 2. Antimicrobial Properties of Plant-Derived Compounds

Quinones are a class of aromatic compounds characterized by a fully conjugated cyclic dione structure; specifically, naphthoquinones are derivatives of naphthalene, where two ketone groups are located on the aromatic ring system [24]. Naphthoquinones, mainly present in Juglandaceae, Lythraceae, Polygonaceae, and Ebenaceae families, have different active compounds such as lapachol and juglone, which exhibit strong antimicrobial properties against *Streptococcus pyogenes* (minimum inhibition concentration (MIC) of 1.56–50 µg/mL) and fungi like *Candida albicans.* They interfere with microbial respiration and DNA synthesis, making them potent agents against various pathogens [25]. In an antibacterial assay, *Enterococcus faecalis* and *S. aureus* were found to be susceptible to thiosemicarbazone lapachol (0.05 µmol/mL), semicarbazone lapachol (0.10 µmol/mL) and juglone, *S. aureus* (32–256 µg/mL), and *L. monocytogenes* (256 µg/mL) [26]. Alkaloids are nitrogen-containing compounds with broad antimicrobial activity, primarily present in Solanaceae, Papaveraceae, Fabaceae, and Rubiaceae [27,28]; examples include yohimbine and vincamine, which are effective against HSV-1 (MIC 0.05–1.6 mg/L) and bacteria like *Vibrio cholerae*. The mode of action (MoA) involves disrupting nucleic acid synthesis and enzyme inhibition [26]. Benzophenanthridine alkaloids from *Chelidonium majus* show activity against MRSA (MIC 0.49–15.63 µg/mL) [29]. Another example studied by Medeiros et al. in 2010 [30], using *Tabernaemontana catharinensis* root bark ethanol extract, proves that its main alkaloids (Ibogamine, Ibogaine, Vocangine, Voachalotine, De-(hydroxymethyl)-Voachalotine, and 12-methoxy-4-methylvoachalotine) have an antimicrobial effect on *S. aureus, Staphylococcus epidermidis, Escherichia coli*, and *Pseudomonas aeruginosa,* with MICs ranging from 20 to 40 µg/mL. Thiosulfinates belonging to the organosulfur compound family are sulfur-containing organic molecules characterized by the general structure R-S(O)-S-R’, where R and R’ are organic groups, mainly present in Brassicaceae, Alliaceae, and Liliaceae. For example, allicin, from garlic and onions, exhibits strong antimicrobial activity, inhibiting *Helicobacter pylori* (MIC 4–6 µg/mL), *E. coli* (MIC > 256 µg/mL), *Salmonella enterica* (MIC > 256 µg/mL), *Yersinia enterocolitica* (MIC > 256 µg/mL), and fungi like *C. albicans* (MIC 64–128 µg/mL) [31,32]. Allicin reacts with thiol groups in enzymes, disrupting microbial metabolism [33,34,35]. Glucosinolates are anions, and the generic chemical structure is composed of a thiohydroximate-O-sulfonate group linked to glucose and an alkyl, aralkyl, or indolyl side chain [36]. Glucosinolates (GS) are primarily from Brassicaceae, Capparaceae, and Resedaceae families [37]. Generically, GS is inactive until hydrolysed into isothiocyanates compounds like allyl-isothiocyanate. Indeed, it is effective against different bacteria such as *E. coli* (MIC 25 mg/L), *P. aeruginosa* (MIC < 1.56 mg/L), and *S. aureus* (MIC 6.25 mg/L) [38]. Another example of antimicrobial glucosinolate-derived nitriles activity is from 1H-indole-3-acetonitrile, 3-phenylpropanenitrile, 4-(methylsulfanyl)-butanenitrile, with MICs of 1.28–2.56 mM for *Fusarium* spp., 2.99 mM for *Aspergillus* spp. and 1.28–1.92 mM against *Penicillum* spp. [39]. The most accredited MoAs are the microbial membrane disruption and protein inhibition [34]. Another important group is iridoids, which are cyclic monoterpenoids that exhibit antibacterial and antifungal properties. Compounds like nepetalactones from *Nepeta* spp. (Lamiaceae family) have MICs of 150–200 µg/mL against *L. monocytogenes* and *S. aureus*, as well as MICs of 200–400 µg/mL against *Bacillus cereus* and *E. coli* [40]. Gentianaceae and Verbanaceae are other vegetal families that have organic compounds and, mainly, anti-inflammatory and anti-haemorrhagic effects, such as gentiopicroside and verbascoside [41,42]. Saponins consist of a steroidal or triterpene hydrophobic aglycone and one-to-three sugar chains (the hydrophilic part) attached via ester or ether linkages [43]. Different saponins are present in Leguminosae, Sapindaceae, and Agavaceae families [44], and they are active against *S. aureus* and *C. albicans* (MICs range from 31.3 to 125 µg/mL). They have also been seen to interact with microbial membrane structures such as glycoside chain length, which significantly influences activity [34,45]. Terpenoids are present in Lamiaceae, Rutaceae, and Pinaceae families and are composed of isoprene units, which encompass monoterpenoids such as thymol and carvacrol, which exhibit significant antimicrobial activity [46,47]. For example, carvacrol has demonstrated minimum inhibitory concentrations of 30 µg/mL against *B. cereus* and *E. coli*, and 15 µg/mL against *Salmonella* Typhimurium and *S. aureus*. In contrast, thymol shows even lower MICs, with 7 µg/mL for *S. aureus, B. cereus,* and *E. coli* and 3 µg/mL for *Salmonella* Typhimurium [48]. These terpenoids are also under investigation for their antifungal properties. Their primary mechanism of action involves disrupting membrane integrity and impairing energy metabolism [49]. Polyacetylenes, commonly found in the Apiaceae, Asteraceae, and Araliaceae family, are characterized by one or more triple bonds (–C≡C–) in their carbon backbone and exhibit antimicrobial, cytotoxicity, anti-inflammatory, and anti-allergic properties [50,51]. (R)-acetoxyfalcarinol and (S)-falcarinol are notable examples of polyacetylenes with antimicrobial properties. These compounds are effective against *B. cereus* and *S. aureus,* with MIC values of 50 μg/mL for (R)-acetoxyfalcarinol and 18.8 and 12.5 μg/mL for (S)-falcarinol [34]. The mechanisms identified include disrupting bacterial membranes and inhibiting intracellular key enzymes [52,53]. Limonoids, a class of tetranortriterpenoids found in citrus fruits, comprise a five-membered lactone ring that is fused to the main tetracyclic triterpenoid framework. Limonoids are notable for their chemical stability and biological activity [54]. Limonoids such as limonin, nomilin, and ekebergolactone are characteristic of plants in the Meliaceae and Rutaceae families and have demonstrated significant antimicrobial activity. Limonoids inhibit different Gram-negative bacteria, such as *Acinetobacter baumanannii* and *Xanthomonas* spp., with MICs between 25–100 and 15.62–62.5 µg/mL, respectively [55,56,57]. The mechanisms seem to be interfering with microbial cell signaling, the mycelian structure, and metabolism [58]. Anthraquinones are naturally occurring organic compounds with a core structure based on anthracene, a tricyclic aromatic hydrocarbon containing two ketone groups. These compounds are found in various plants, such as Rheum species (Polygonaceae family), Asphodelaceae, and Fabaceae, and are recognized for their antimicrobial and antifungal activities [59]. Notable examples of anthraquinones include emodin and rhein, the latter being effective against Gram-positive and Gram-negative bacteria like *S. aureus* (MIC 7.8–31.25 µg/mL), *Streptococcus mutans* (MIC 200 µg/mL), *S. enterica* (MIC 250 µg/mL), and fungi such as *C. albicans* and *Aspergillus fumigatus* (MICs 50 and 25–250 µg/mL, respectively) [60,61,62]. Their mode of action includes disrupting microbial membranes, biofilms, and inhibiting DNA synthesis [34]. Therefore, it is possible to state that literature data are difficult to compare due to the use of various methods for assessing antimicrobial activity, different solvents, and the origin and purity of test compounds, which are often isolated from various plant extracts, but it is possible to affirm that there is a wide group of bio-active compounds of profound interest for human health and foodborne control [1].

### 2.1. Antimicrobial Activity of Polyphenolic Compounds

The antimicrobial activity of PPs is mainly influenced by their chemical structures, such as the hydroxyl group (-OH), the prenyl group (-C_5_H_9_), and the methoxy group (–OCH_3_), as well as the presence of single or multiple glycosylations. Secondly, the antimicrobial activity depends on how the π bond is delocalized on the C-ring [63,64]. Thus, the number and position of substituents, intramolecular hydroxyl cyclization, and the steric hindrance within the molecule affect the antimicrobial activity of PPs [64,65]. For example, 5,7-dihydroxy-4′,6,8-trimethoxyflavone (C_18_H_16_O_7_) exhibits activity against *Escherichia coli* and *Staphylococcus aureus*, while 5,6-dihydroxy-4′,7,8-trimethoxyflavone (C_18_H_16_O_7_) lacks apparent antimicrobial activity against these microorganisms. This difference is due to the different positions of a methoxy and hydroxy substituent [66]. Moreover, glycosylation can affect antimicrobial activity, as seen in comparisons between quercetin-3-*O*-rhamnoside and quercetin, because glycosylation can reduce the antimicrobial activity of quercetin. Thus, quercetin alone, without being glycosylated, exhibits a stronger effect on *C. albicans*, *B. subtilis*, *S. aureus*, and *E. coli* [63]. Previous studies have reported that various PPs, such as quercetin, have fewer free-OH groups than other phenolic compounds, which increases the chemical affinity for the microbial lipid membrane because they are less hydrophilic. Thus, it has been assessed that they possess stronger antimicrobial activity [67,68]. The hydroxyl group ensures that molecules can interact by means of hydrogen bonds with RNA and DNA, proteins, and other charged molecules. Indeed, the aromatic backbone structure can relocate electrons, and their stability also depends on the different substituents, affecting the lipophilic behaviour and, consequently, the antioxidative or oxidative behaviour (on foodborne pathogens, in food matrices, and in human health) [3]. The antimicrobial properties of bound PPs, and other compounds such as sterols, glycosides, and pentacyclic triterpenoids from the non-extractable fraction in different fruits and vegetables, such as pomegranate peel, which is commonly known to be very rich in non-extractable polyphenols (NEPPs), have not yet been deeply investigated. Due to their bound condition, the IBPs arrive in the low intestine, inducing a prebiotic effect in addition to a positive interaction with the environment, enhancing their bio-accessibility through fermentation [69]. In contrast, free PPs have a more direct effect and bioavailability [70]. It has been demonstrated that different plants have different PPs, so the prebiotic effect can enhance different beneficials. In the same study, concerning the Rosaceae family, Zhou et al. [70] showed that dietary PPs shape the composition of the gut microbiota, with *Akkermansia* and *Ruminococcus* helping to maintain the mucus structure and barrier. Lee et al. [71] proved that with blueberry supplementation in the diet of rats, the growth of Gammaproteobacteria was stimulated. Yan et al. [72] demonstrated a similar effect, showing that anthocyanins from *L. ruthenicum* increased the relative abundances of *Bifidobacterium* and *Allisonella*, two important beneficial bacteria genera. In the past 20 years, research has aimed to isolate polyphenolic compounds from NEPP extracts. Gonzàlez–Sarrìas et al. [73] have shown that the non-extractable fractions of various fruits, such as pomegranate peels, possess multiple phenolic hydroxyl groups, making their structures and chemical properties highly unstable. This instability arises from their susceptibility to oxidation and degradation during extraction and purification processes. The quality of NEPPs depends on their vulnerability to environmental factors, such as raw material stability, storage conditions, and, more specifically, temperature, oxygen levels, pH, and light. These changes reduce the antimicrobial potential and antioxidant ability of these compounds in various matrices and substrates, as well as their benefits for human health [69].

### 2.2. Interaction Between Polyphenols and Foodborne Bacteria

**Figure 2:**
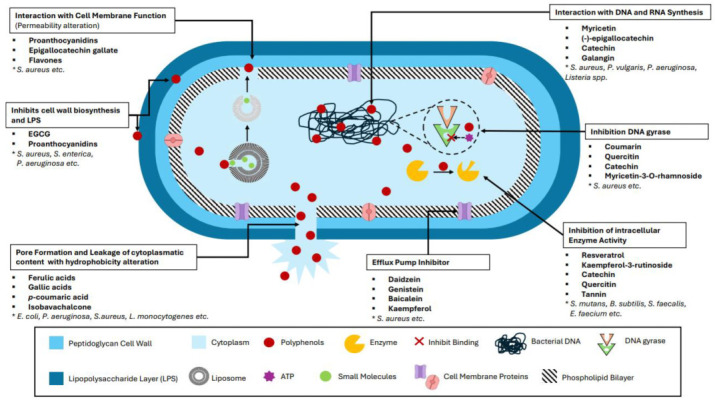
Proposed mechanisms of polyphenols as antimicrobials. Adapted by [74]. The figure has been drawn using BioRender site.

Depending on the molecular structure of the different phenolic classes and the environment, antimicrobial activity can occur through various mechanisms, including (i) a direct interaction with the cell membrane, leading to its disruption or structural modification; (ii) a reduction in the expression of certain genes, making the cell incapable of producing specific proteins for quorum sensing; (iii) a direct influence on DNA and RNA and some proteins essential for the cell; (iv) metal ion complexation chelating metals, which are needed by bacterial proteins and required for membrane depolarization, attacking the productivity of ATP synthase; and (v) a direct interaction with the cell metabolic pathways, reducing cell efficiency and ultimately leading to bacterial death (Figure 2).

#### 2.2.1. Polyphenol Interaction with the Microbial Cell Membrane

The interaction between PPs and the microbial cell can lead to cell membrane damage or cell death (Figure 2). Fan et al. [75] and Zhang et al. [76] have demonstrated the role of epigallocatechin gallate (EGCG) and other PPs in inhibiting bacterial fatty-acid synthesis and fatty-acid elongation by affecting FabG and FabI enzymes, disrupting the membrane and preventing FabI from binding the nucleotide cofactor NADH. Gòrniak et al. [77] and Karas et al. [78] revealed that the presence of galloyl groups significantly increased hydrophobicity and the affinity to lipid bilayers. Thus, catechins, more than other PPs, can influence membrane structure by intruding into the hydrophobic centre of the double-phospholipid membrane. Wang et al. [79] noted that the antibacterial activity of catechins and their affinity to lipid bilayers increased with the number of carbon atoms in their alkyl chain; specifically, the most potent antibacterial activity is observed when the alkyl chain consists of 4–7 carbon atoms. The same study reported that EGCG causes damage to the *E. coli* cell membrane, such as nanoscale perforations. Furthermore, the same molecules create microscale grooves in the cell wall and aggregates in the cell envelope of *S. aureus*, leading to cell lysis. Wang et al. [80] determined that the deterioration of the cell walls of *S. aureus* is likely caused by EGCG binding to the peptidoglycan layer, while in *E. coli*, cell wall damage results from oxidative stress and the production of H_2_O_2_. These results prove that the mechanisms of antibacterial activity against Gram-positive and Gram-negative bacteria differ [81]. Due to the high specificity of each compound and the many different cross-interactions, Konatè et al. [82] stated that the resistance of Gram-negative bacteria to polyphenolic compounds is due to the lipophilic nature of their outer membrane, which contains high levels of phospholipids and enzymes in the periplasmic space. On the other hand, Plumed–Ferrer et al. [83] suggested that various PPs can disintegrate the outer membrane of Gram-negative bacteria, thereby enhancing membrane permeability.

#### 2.2.2. Polyphenols and Their Impact on Microbial Proteins, DNA, and RNA

Generically, all PPs are composed of polyhydroxy compounds consisting of hydroxyl groups and a hydrophobic benzene ring, which enable them to interact with proteins, DNA, and RNA through amino compounds and carboxyl groups or hydrophobic interactions [84]. The B rings of flavonoids can inhibit DNA and RNA synthesis by hydrogen bonds with nucleic acid bases of bacteria or intercalation [74]. In addition, as seen in other studies, catechin derivatives, especially EGCG (but also quercetin), have an inhibitory effect on bacterial DNA gyrase (Figure 2) [74,85]. Indeed, Ulrey et al. [86] have demonstrated that *P. aeruginosa* treated with cranberry proanthocyanidins had a decreased expression of proteins linked to ATP synthase, cytochrome c protein, DNA and RNA synthesis, and components of the citric acid cycle. At the same time, an upregulation of various proteins was observed, including those associated with cation transport, amino acid biosynthesis, iron siderophores, and stress-response proteins. Also, membrane-transport proteins, such as ATP-binding cassette transporters (ABCs) and solute carriers (SLCs), have been found to interact with many PPs [87]. Furthermore, the interaction of PPs with various transporters that are responsible for eliminating potentially toxic products deprives bacteria of one of the most important mechanisms for exporting dangerous molecules, reducing bacterial efficiency [4].

#### 2.2.3. Polyphenols, Metal Ion Complexation, and Membrane Depolarization

The presence of metals, whether within heme proteins or independently, stands as a determinant factor of oxidative stability in foods (Figure 2) [88]. The synergy between polyphenols and metal ions enhances antimicrobial activity through various mechanisms: (i) metal ions stabilize polyphenol structures, improving interactions with microbial membranes and enzymes; (ii) these complexes inhibit microbial enzymes, generate reactive oxygen species (ROS) that damage cellular components, and alter membrane permeability to facilitate polyphenol uptake, (iii) additionally, metal ions increase the binding affinity of polyphenols to microbial targets, amplifying their ability to inhibit growth and disrupt metabolism [89]. Indeed, it has been indicated that the presence of metals accelerates the oxidation process in some matrices more effectively than temperature or light [88,90]. The oxidation state of PPs influences the interaction between trivalent iron and oxygen within the substituent group positions of PPs. This interplay can create a microenvironment with limited availability of reagents for biological reactions, where trivalent iron or other minerals serve as a critical cofactor for many common enzymes, acting as limiting reagents; for example, different PPs (like catechins) can bind metal ions (such as copper, iron, or zinc), reducing the metabolic efficiency of specific bacterial strains, depending on the PPs used, and another study demonstrated that myricetin, quercetin, and rutin, along with epicatechin, exhibited greater oxidative stability compared to the synthetic antioxidants evaluated [91]. Gust and Wawer in 1995 [92], studying radical scavenging, proved that one or two oxygen ions (depending on the electronic delocalization due also to the phenolic group) can interact with trivalent iron, thus being noticed for their binding, antioxidant capabilities. Furthermore, Zhang et al., in 2021 [84], demonstrated that a specific siderophore (from *Ustilago sphaerogena,* fungi) can be less efficient than different catechins (like EGCG) in iron-chelating. Metals are determinant, not only for a various spectrum of metabolisms, but also because they impact the bioavailability of essential minerals and induce chemical modifications in different organic molecules, such as amino acids [93,94]. Heat treatments significantly reduce the levels of soluble and dialyzable zinc (Zn) and iron (Fe), primarily due to their interactions with reducing sugars and amino acids. Research shows that Maillard reaction products hinder the absorption of Zn, Fe, and copper (Cu) in both laboratory settings and live models, with crude glucose–glutamate reaction products exhibiting stronger metal-binding capacities compared to fructosyl-glycine. Notably, the hierarchy of metal-binding strength—Mg^2+^ > Cu^2+^ ≈ Ca^2+^ > Zn^2+^—diverges from the Irving–Williams order, indicating the involvement of diverse ligand types [91]. Factors like cation species, amino compound characteristics, and reaction conditions influence the chelating properties of Maillard reaction products, the stability of their complexes, and the formation of chromophores [95]. Furthermore, in food preservation, the use of PPs in the presence of transition metals, such as copper (Cu(I)) or iron (Fe(II)), may result in the formation of hydroxyl radicals (•OH) from H_2_O_2_ adding another layer of complexity to these chemical interactions [91,96]. Metals are also present in prooxidant enzymes, such as peroxidases, dioxygenases, and lipoxygenases, which are slowed down by PPs and compete with them, depleting the environment of cofactors such as iron or copper [97]. Dadi et al. [98] showcased the inhibitory effect due to membrane depolarization of polyphenolic compounds on ATPase activity and ATP synthase in *E.* coli. Piceatannol completely inhibited ATPase, quercetin for 80% (IC50 = 33 μM), quercetin-3-β-D-glucoside for 50% (IC50 = 71 μM), and resveratrol for 40% (IC50 = 94 μM). Stilbenes, resveratrol, and piceatannol suppressed ATPase and ATP synthesis, while the other compounds only inhibited enzyme activity. The same capability of affecting the cell membrane potential and the sodium–potassium ATPase pump, thus altering ATP synthesis, has also been seen in phenolic acid, with the different structures of this group potentially inducing hyper-acidification at the plasma membrane [99].

#### 2.2.4. Polyphenols Interfering with Cellular Metabolism and the Efflux Pump

PPs also have the ability to impact the biosynthesis of proteins within bacterial cells, thereby altering their metabolic processes (Figure 2). It is known that flavonoids, generically, inhibit fatty-acid synthase, and condensed tannins exhibit a strong affinity to the outer membrane of *B. subtilis*, *S. aureus*, and *E. faecalis* [74]. For example, quercetin and kaempferol-3-rutinoside (nicotiflorin) inhibit sortases A and B, which are indispensable enzymes for protein synthesis, mainly for virulence and storage, in *S. aureus* and *S. mutans.* The same study showed that PPs can be used to enhance antibiotic efficiency, aiding in the treatment of antibiotic-resistant bacterial infections. For example, quinones and chalcones, which can strongly interact with bacterial efflux pumps, are used with efflux inhibitors to increase drug accumulation in cells by altering transport through the cell wall and the phospholipid bilayer [1]. Bae et al. [74] have demonstrated that when treated with galangin, *S. aureus* exhibited a loss of approximately 21% of cytoplasmic potassium ions. This efflux of potassium ions through the outer membrane prevents cytoplasmic acidification and the subsequent formation of destructive lesions. In a related study, Silberberg et al. [100] showed that galangin inhibits potassium-induced mitigation of cellular damage through Kdp-FABC (the ABC superfamily is one of the seven principal families of efflux pumps). Another example has been reported by Bame et al. [101], who showed that flavonoids from *Alkanna orientalis* (Boraginaceae family) inhibit NorA efflux pump activity in *S. aureus,* leading to cell death. Furthermore, controlling the efflux pump efficiency partly influences metabolism, thereby also restoring the conventional effectiveness of various antibiotics against their resistant bacteria. For example, different kinds of PPs, such as baicalein, and two types of triterpenic acids have been shown to restore the efficiency of tetracycline and methicillin against methicillin-resistant *S. aureus* (MRSA) [102].

#### 2.2.5. Interaction Between Polyphenols and Biofilms

One important ability of PPs is to interact with quorum sensing and biofilms. The effects of several phenolic compounds on biofilm formation by *P. aeruginosa* have been investigated, showing that, depending on their concentration, PPs can have either an inhibitory or a stimulating effect on biofilm formation [103]. At lower concentrations, which did not inhibit bacterial growth or only weakly inhibited it, the compounds enhanced biofilm formation. For example, vanillin, epicatechin, gallic acid, and cinnamic acid have opposite effects depending on their concentrations. Procyanidins, on the other hand, exhibit anti-biofilm activity by interacting with the essential p-fimbriated adhesion of *E. coli,* which is crucial for biofilm development, decreasing its hydrophobicity and, consequently, reducing its proliferation [104]. Majidinia et al. [85] observed that cranberry proanthocyanidins exerted inhibitory effects on the swarming motility of *P. aeruginosa* and influenced its biofilm formation. At low concentrations of 1 mg/mL, the inhibition rate reached 40.9%, whereas at a higher concentration of 10 mg/mL, the inhibition rate increased to 55.7%, with a reduction in the thickness of the biofilm of 26 to 20 μm (23% thinner). This decrease in biofilm density appears to be related to significant differences in the expression of 159 proteins. Certain bacteria can redirect energy toward increasing cell density, as detected by quorum sensing, when polymer production in biofilms is inhibited. This shift is reversible, and enhanced polymer secretion improves environmental sensing under low cell density conditions. Such adaptability highlights the interplay between biofilm formation and environmental perception [105].

## 3. Synergistic Effects of Polyphenols

Synergism in drug combinations refers to the interaction of two or more compounds that result in an effect greater than the sum of their individual effects. Such combinations enhance drug efficacy while reducing the required doses, minimizing potential toxicity [106]. A multitarget approach using synergistic combinations can also help counteract drug resistance development [107]. The synergistic effect is assessed using the Fractional Inhibitory Concentration Index (FICI), a quantitative measure of interaction between two antimicrobial agents. A FICI ≤ 0.5 indicates synergy (optimal interaction), 0.5 < FICI ≤ 1 represents an additive effect, 1 < FICI ≤ 4 suggests indifference, and FICI > 4 denotes antagonism [108,109]. For instance, combining oregano essential oil (OEO) at 0.2% with caprylic acid (0.5%) and citric acid (0.1%) in vacuum-packaged beef reduced lactic acid bacteria by 1.5 log CFU/g and *Listeria monocytogenes* by over 2.5 log CFU/g compared to the control [109]. This effect is attributed to the volatile organic compounds (VOCs) released by OEO, primarily carvacrol, *p*-cymene, and thymol, which effectively target foodborne pathogens [110]. Another example involves antibiotics: the minimum inhibitory concentration MIC of tetracycline against *Salmonella* Typhimurium decreased from 160 μg/mL to 40 μg/mL when combined with lariciresinol, a lignan compound [111,112]. Owen and Laird (2018) [113] demonstrated that antibiotics could exert synergistic effects with PPs by targeting specific bacterial functions. A similar study on pomegranate peel extracts against Multi-Drug-Resistant (MDR) *Salmonella enterica* serovar Typhi, serovar Typhimurium, and *E. coli* showed inhibitory activity due principally to punicalagin (an ellagitannin). Kiran et al. (2024) [114] compared the efficacy of punicalagin, methanol extracts of pomegranate peel (PPME), and antibiotics alone or in combination. The MIC values of PPME were 2.5–7.8 μg/mL for *E. coli*, 3.9 μg/mL for *S. typhi*, and 7.8 μg/mL for *Salmonella* Typhimurium. When comparing PPME with antibiotics (i.e., aminoglycoside, β-lactam, and fluoroquinolone) against antibiotics alone, there is an increase in inhibition zone values, varying from 3.4 ± 2.7% to 73.8 ± 8.4%. In conclusion, interactions between organic compounds and antimicrobial agents—both natural and synthetic—can significantly enhance antimicrobial activity. Certain compounds may synergistically improve the properties of others, either individually or within consortia, although these interactions may occasionally augment the antioxidant capacity of specific bacterial strains, reducing their antimicrobial effects [115]. Further studies are needed to elucidate these mechanisms. Future research should focus on computational modeling to predict promising chemical structures and on biological studies to establish the No Observed Adverse Effect Level (NOAEL), improving our understanding of polyphenol–microbe–human health interactions [116]. For example, the open-source ToxDP2 data repository has assembled toxicological, biological, and chemical information on 415 dietary polyphenols, incorporating 25,792 data points to assess their safety in food applications. Although certain knowledge gaps persist, this resource offers valuable perspectives on food safety and risk evaluation of polyphenols. Accordingly, despite their well-documented antioxidant and anti-inflammatory properties, apprehensions endure regarding their potential toxicity, including carcinogenic and genotoxic impacts. The variation in effectiveness and toxicity hinges on molecular configuration, interactions, and individual reactions to dietary conditions. Consequently, no universally recognized safety thresholds exist for specific polyphenols. Therefore, limited toxicity assessments of food and feed products can generate concerns about the hazards linked to high-dose exposure. Although outside the scope of this narrative review, the evaluated scientific literature revealed NOAEL values strongly dependent from each phenolic structure (ranging from 5 up to 2000 mg/kg/day). Therefore, a better understanding of these fluctuations is crucial for evaluating the therapeutic and, eventually, toxicological potential of polyphenols and for guaranteeing the safe utilization of dietary supplements.

## 4. Potential Application of Polyphenols in Food Systems and Their Sustainability

To enhance the functionality of polyphenols and meet consumer demands for green-based products, there are various possibilities [117]. Using different coatings, enriched with a wide range of organic molecules (OM) (for example, using PPs like tannic acid), it is possible to decrease the viability of *P. aeruginosa* and *S. aureus* by at least 30-fold upon exposure [118]. In the same study, Sileika et al., in 2013 [118], assessed that mixing tannic acid and pyrogallol did not exhibit harmful effects, cytotoxicity, or other health issues, an area of study extensively explored in various investigations [119,120,121]. Clearly, it is also critical to demonstrate the antioxidant potential of polyphenols through coatings that preserve their functionality over time and under various conditions [122]. This stability can be achieved through different approaches, primarily by protecting the polyphenols from biotic and abiotic stressors such as light, heat, pH variations, mechanical stress, digestion, and fermentation [123,124]. The carrier used to deliver this positive effect can significantly alter the final outcome. Indeed, Fu and Dudley in 2021 [125] demonstrated that coatings can also have positive physiological effects, further enhancing the potential of polyphenols. Environmentally, the growing problem of non-biodegradable plastic waste has driven efforts to develop renewable biopolymers, such as pectin, a plant cell wall polysaccharide [126]. Pectin is biodegradable, non-toxic, water-soluble, and excels in film formation, making it an ideal candidate for active food packaging. Its ability to bind polyphenols and other beneficial molecules aligns with circular economy principles [127,128]. Lima et al. (2022) [129] demonstrated that edible coatings on vegetables and fruits were able to reduce up to 2 log CFU/g of aerobic mesophiles on apples (for example, after 15 days of storage) and minimize the enzymatic browning. Regarding the delivery of antimicrobial/antioxidant compounds, polysaccharides (such as chitosan and pectin, which are often supplemented with low-density proteins to improve technological performance) are commonly used [124,125]. However, this can impact another critical aspect: the compounds can alter the physical properties of the film. Depending on the formulation, the original composition of the polysaccharide, and the type of polyphenols selected, the final outcome can vary. For instance, different combinations of carbohydrates and polyphenols, such as film-based curcumin used as freshness monitoring or, in other carriers for its antioxidant activity [130] and EGCG for its antioxidant and antimicrobial properties, can result in distinct outcomes [131]. These combinations may generate various substructures that influence not only macromolecular behaviour but also microscopic characteristics, ultimately impacting antimicrobial and antioxidant potential. The broad spectrum of efficacy may further vary depending on the concentration of the active molecule [127,132]. In fact, for various films, it is essential that the polysaccharide does not bind covalently to maintain optimal bioaccessibility and preserve its properties or, conversely, bind covalently depending on the desired effect [133,134]. Furthermore, in a general overview, there is also the direct application of organic molecules like EO [135], polyphenols [23], organosulfur compounds, and peptides [136]. This method is facilitated by its ease of application, even when these compounds are not protected from environmental factors, which reduces their overall efficacy [137]. However, enhancing the bioactive profile is crucial, as the NEPP profile might remain unchanged, potentially overlooking this portion of polyphenols [138]. In this case, the efficacy is influenced by the high complexity of interactions between the environment, food matrix, and technological processes [139,140,141]. Moreover, direct application can lead to non-uniform distribution, causing localized over- or under-activity, thus reducing efficiency [142].

The incorporation of different types of organic molecules, such as essential oils, into food products can dominate the flavour, potentially reducing palatability and consumer acceptance. To this aim, different formulation technologies have been implemented, such as the utilization of masking agents and/or synergistic molecules [143,144]. Another interesting and deeply studied modality of delivery is nanoencapsulation (NE), which is emerging as a promising strategy to augment and optimize bioaccessibility and protect polyphenols from environmental factors [145]. This can be done, for example, by carrying polyphenols with different minerals or molecules or incorporating them into nanoemulsion-based carrier systems [146]. Regarding nanoencapsulation, microemulsions are less effective in terms of organic molecules delivered and available ingredients for emulsification, such as emulsifiers and oils, as reported by McClements in 2012 [147] (due to the small hydrophobic domain available). Nanoencapsulation has a reduced droplet size, offering numerous advantages compared to traditional emulsions. Mainly, it is possible to control the dispersion over time due to the release kinetics, which is also related to the surface area of the droplet. Furthermore, nanoencapsulation minimizes aggregation and sedimentation risks while maintaining the correct ratio of interaction in environments with different polar behaviours [145,148]. However, challenges remain. As assessed by McClements [149], depending on the different nanomaterials or chemical compounds (even in trace amounts) used, this may lead to gastrointestinal dysfunction, thus reducing the efficacy of delivery. Additionally, there is the possibility of denaturation of different enzymes when in contact with nanomaterials, leading to a decrease in catalytic activity [150] and functional damage to microvilli and tight junctions between epithelial cells, reducing the normal capability of nutrient absorption [151]. Furthermore, any antimicrobial that is not chemically degradable can accumulate in various tissues or organs through ingestion [152]. This uptake and accumulation depend primarily on the physicochemical characteristics of the nanomaterials, such as morphology, composition, and size, as reported by McClements et al. [153]. Focusing on the microbiological perspective, Rahmani et al. [154] compared different hydroethanolic extracts with NE and nanoemulsion (NM), showing inhibition on *K. pneumoniae* and *Salmonella* Typhimurium, 78.5% and 73%, respectively, proving that with different extracts, it is possible to have a similar effect between NE and NM, thereby promoting the utilisation in the food industry. Another study analyzes the effect of different natural antimicrobials, either in NM form or free form, demonstrating the higher efficiency of NE essential oils, significantly changing their MIC (evaluated as inhibition halo) from 48% to 72% for *E. coli* and from 30% to 72% for *Salmonella* Typhimurium [155]. Moreover, in the same study, NE containing different concentrations of EO has shown reduced *S. aureus* vitality from 50% to 100%, compared to free form. Instead, on *L. monocytogenes*, the free form is more effective than the delivered one. This is a direct consequence; when the compound is in a delivery system, it needs time to cross the membrane and enter in contact with the microorganism [156]. Indeed, nanoencapsulation is emerging as a promising method to protect phenolic compounds and other bioactives from degradation, ensuring targeted delivery with optimal bioactivity [157]. The high correlation between bioactivity and efficacy as an antioxidant and/or antimicrobial is clear, but due to the strong synergy among different polyphenolic classes, there are few targeted quantitative studies available. The limited research conducted primarily focuses on antimicrobial extracts in the context of food preservation with or without NE, which are often poorly characterized from a chemical and structural standpoint. Thus, even though some oxidative pathways are defined, each PP has a different MoA to interact with other PPs, carriers, and with the matrix [158]. As a general consideration, evaluating the risks associated with nanostructures can provide valuable scientific insights into their safety and potential toxicity while also offering guidance on their application in food products.

Looking at the extraction process and its sustainability, it is possible to state that largely depends on the methodology employed; for example, in solvent-assisted extraction, the key optimization parameter identified by LCA findings is minimizing solvent quantities [157]. However, the efficiency of this method is heavily influenced by the original matrix, as it often results in partial extraction and fails to recover a significant portion of non-extractable polyphenols (NEPPs) [158]. The primary concern with this approach lies in the use of organic solvents, which cannot be completely removed from the substrate, are non-recyclable, and require multiple stages throughout the extraction process [159]. Consequently, due to stricter regulations and health concerns, alternative technologies are being explored, such as enzyme-assisted extraction. This method is highly efficient and specific, albeit sophisticated and less intuitive yet eco-friendly and more effective [160]. It relies on the hydrolytic degradation of cell wall polymers, which is crucial as many polyphenols form complexes with organic molecules like proteins or carbohydrates, making their extraction—particularly of NEPPs—more challenging [159]. Studies by Dominguez–Rodriguez et al. [161] and Swer et al. [162] have demonstrated that enzyme-assisted extraction positively impacts the extraction of proanthocyanidin fractions from *Prunus avium*, enhancing total polyphenol content (TPC) and quality while increasing bioactivity compared to other methodologies. Similarly, Zhang et al. [163] showed that microwave-assisted enzymatic extraction of waste peanut shells could increase polyphenol content by 62.73% while maintaining antioxidant and antibacterial properties. These findings highlight the importance of tailoring extraction methods to the specific molecular structure and interactions of each polyphenol class. In conclusion, considering that an average of 45% of fruits and vegetables produced for human consumption are discarded as residues and by-products across the food supply chain, there is significant potential to repurpose this biomass within a bio-circular economy [164]. Developing scalable and specific extraction methods for the most commonly wasted fruits and vegetables represents a promising future direction.

## 5. Description and Classification of Flavonoids

### 5.1. Flavonoids

Based on the saturation and oxidation level on rings A and B, the flavonoids in food can be grouped into different subclasses and categories (Figure 1). The 43 distinct substructures of PPs and flavonoids are grouped into six subclasses: (i) flavones, (ii) isoflavones, (iii) flavonols, (iv) flavanols, (v) flavanones, (vi) flavanonols, and (vii) chalcones [1,4,6]. Furthermore, Yuan et al. [8] proved that the molecular system that allows flavonoids to protect biological structures can be attributed to their antioxidant activity due to their double capability to donate a hydrogen atom and electron-withdrawing groups. This ambivalence is a positive factor for antibacterial activity [165]. Moreover, by analysing the chemical structure, Tian et al. [166] have proved that antioxidant and antimicrobial efficacy are directly correlated with the number of phenolic hydroxyl groups. Comparing anti-inflammatory and antioxidant activities, Yuan et al. [8] revealed that compounds containing enol groups (flavonols and chalcones) exhibited superior properties compared with those lacking enol groups. Flavonoids have an important potential role in physiological regulation, chemical signaling, and responses to stress and pathogens. They are synthesized in all parts of the plant, and their distribution in vegetables is mediated by several factors, such as water availability, light, and stress [167,168]. In humans, in particular, flavonoids have been demonstrated to play a relevant role in cardioprotection by preventing LDL oxidation, exhibiting antiapoptotic and antinecrotic effects, scavenging free radicals, and inhibiting the propagation of inflammation. However, an overdose of polyphenols (PPs) has been shown to reduce the bioavailability of metals, vitamins, and other antioxidants. [169]. Different studies have demonstrated that a diet rich in polyphenolic compounds and using them in nutraceutical products improve human health [13]. The absorption of PPs depends on their chemical structure. Only flavonoid aglycones and a few glucosides can be absorbed in the small intestine, while the remaining PPs (IBPs) are degraded by microorganisms in the colon into phenolic acids [170,171,172].

#### 5.1.1. Flavones

**Figure 3:**
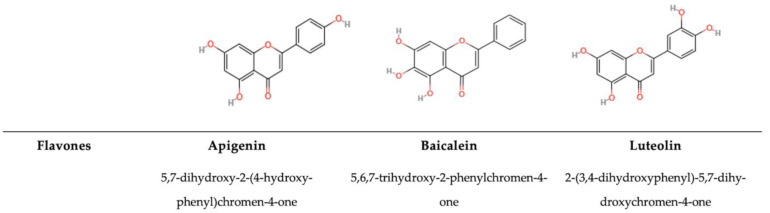
Chemical structure of some representative flavones and their respective IUPAC names. The molecules were represented using the MolView application (version 2.4).

Flavones are structurally very similar to flavonols. They differ in a substituent in the C2 position, with OH for flavonols and H for flavones, and they contain a C2′–C3′ conjugated bond and a C-ring, which holds a ketone group at the C4′ position (Figure 3) [1]. Luteolin, baicalein, tangeretin, and apigenin are the most common flavones found in food, such as red pepper, grapes, celery, and onions [137,173]. These compounds have a wide range of functions in biological systems, including anti-inflammatory and anti-carcinogenic effects [174]. For example, luteolin can interact with Gram-positive and Gram-negative bacteria, such as *B. cereus, B. subtilis, E. faecalis, S. aureus*, *P. aeruginosa, *and* E. coli,* with MIC ranging from 19 to 156 μg/mL [175]. Karpinski et al., in 2020 [176], demonstrated that orientin, isoorientin, chrysin, apigenin, and isovitexin have antimicrobial power on *E. faecalis, E. coli, P. aeruginosa,* and *S. aureus*, with MICs ranging between 500–1000 μg/mL. Another study focused on the antimicrobial potential of glycosylated flavones, demonstrating that luteolin-7-O-glucoside and apigenin-7-O-glucoside inhibit the growth of various bacteria species such as *E. coli*, *B. cereus, E. faecalis, S. aureus*, and *S. typhi*, at multiple levels of inhibition, bonding different enzymes [177]. Comparing the efficiency of different PPs, both glycosylated and non-glycosylated, it has been observed that in the flavone group, apigenin, which inhibits *E. coli*, can interact with different enzymes, more so than the glycosylated form, such as DNA gyrase and proteins involved in biofilm formation [178,179]. Instead, quercetin, enhances cytoplasmatic membrane permeability in *Streptococcus pyogenes* at a concentration of 128 µg/mL, leading to an inhibitory effect [180]. Another PP able to interact with the membrane is vitexin, which decreases the hydrophobicity of the cell surface and alters the membrane permeability of *S. aureus* at the sub-MIC concentration of 126 µg/mL. Other flavones, such as isovitexin, decrease the adhesion of MSSA but enhance the adhesion of *E. coli* at MIC 200–500 µg/mL [181,182]. Another study enriching the knowledge of different MoAs involved by Adamczak et al. [183] compared flavone, luteolin, apigenin, and chrysin, showing that hydroxyl groups on phenyl rings A (C-5, C-7) and B (C-3′, C-4′) generally had no impact on the flavones’ antimicrobial activity.

#### 5.1.2. Flavonols

**Figure 4:**
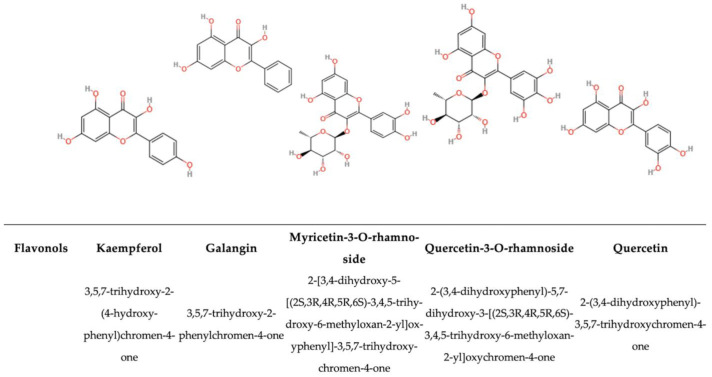
Chemical structure of some representative flavonols and their respective IUPAC names. The molecules were represented using the MolView application (version 2.4).

Flavonols (FLs) are a subclass of the flavonoid family (Figure 4). However, they differ from flavones in that they contain a hydroxyl group at the C3 position [184,185], an oxygen group at the C2 position, and a 2,3-double bond on ring B, which allows conjugation between rings A and B and strongly affects the redox properties of these compounds [186]. It has been theorized that the B-ring substitution type is a decisive factor in determining the antiradical effectiveness of flavonoids, and that the A-ring substituents are not directly engaged in the scavenging process [3]. The most well-known flavonols (in aglycone form) are quercetin, myricetin, and kaempferol. The richest sources of FLs are onions, reaching 1.2 g/kg fresh weight, while red wine has the minimum level of FLs, reaching 2 mg/L [138]. In descending order of the amount of FLs, curly kale, broccoli, and leek are also rich in FLs [186]. These flavonols usually accumulate in organs and tissues where light has a direct impact. The biosynthesis of each PP in plants follows a different pattern; the biosynthesis of FLs is indeed mainly stimulated by light. As has been seen in lettuce and cabbage (leafy vegetables), the glycoside concentration in the external leaves can be up to 10 times higher than that in the inner leaves, which are typically light-coloured [187]. Identifying common catabolic pathways for PPs in the gut microbiota population through different families of bacteria, such as quercetin degradation via taxifolin and alphitonin by *Eubacterium ramulus* and *Clostridium orbiscindens*, also enables the study of secondary metabolism, highlighting possible crossed interactions among bacteria that may be related to total polyphenol content [188]. The same analysis can be done with foodborne pathogens. Indeed, quercetin and naringenin exhibited mild-to-strong antimicrobial activity against *S. aureus*, *S. epidermidis, B. subtilis, E. coli,* and *P. aeruginosa* [189]. Specifically, quercetin was found to bind the 24 kDa fragment of the gyrase B subunit of the bacterial enzyme DNA gyrase, which is essential for the negative supercoiling of DNA during replication or transcription. Additionally, quercetin is a potent anti-inflammatory and antioxidant agent, with promising potential as an adjuvant treatment for inflammatory diseases and oxidative stress. It supports human health also inhibiting harmful bacteria while promoting a healthy environment [166].

#### 5.1.3. Isoflavones

**Figure 5:**
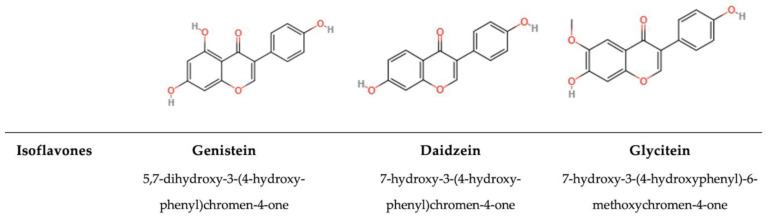
Chemical structure of some representative isoflavones and their respective IUPAC names. The molecules were represented using the MolView application (version 2.4).

The chemical structure is based on a C15 carbon atom skeleton composed of two aromatic rings and one heterocyclic ring (Figure 5). One aromatic ring (the A ring) is condensed with the heterocyclic one (the C ring), and the third ring (the aromatic ring B) is connected to the A and C rings. The B ring in isoflavones is attached to C3, whereas in flavones, it is attached to C2 [184]. Although natural sources of isoflavones include the Fabaceae family, red clover, alfalfa, kudzu, and species of the genus *Genista* [190], one of the most important crops for isoflavones, historically and chemically, is soybean. Before 2000, plant-derived natural products of isoflavones were mainly confined to the Leguminosae family [191]. Soybeans contain 12 kinds of isoflavones, depending on the type of aglycone and functional group [192]. Soybeans have an isoflavone content ranging from 580 to 3800 mg per kilogram of fresh weight, whereas soymilk contains between 30 and 175 mg/L [193]. In humans, isoflavones show structural similarity to the female hormone. Because of this similarity, they are able to bind estrogenic receptors and have similar biological activities. Therefore, they are called phytoestrogens [190]. Apart from their hormone-like structure, isoflavones are abundant in foods. After isoflavone metabolization in the human gut by gastrointestinal enzymes, the precursors genistin and daidzin become the aglycones genistein and daidzein, respectively [194]. Isoflavones have been demonstrated to aid in the prevention and management of various dysfunctions and ailments associated with aging, encompassing neurodegenerative conditions, osteoporosis, metabolic and cardiovascular disorders, and manifestations of menopause [190,191,192,195]. For example, genistein, the most extensively researched soy isoflavone, is recognized for its ability to inhibit the proliferation of *Bacillus anthracis*, *Bacillus cereus*, *H. pylori*, methicillin-resistant *S. aureus* (MRSA), *Streptococcus pyogenes*, and *Vibrio harveyi* [195,196]. However, it is ineffective against *E. coli*, *K. pneumoniae*, *L. reuteri*, and *Shigella sonnei*. Conversely, daidzein has a high bioconversion value and shows significant inhibitory effects against *S. aureus*, *E. coli*, *S. typhi*, and *L. monocytogenes* [197]. Furthermore, various bacteriostatic molecules have been identified. For example, the methanolic extract of doenjang (MED) contains an isoflavone with high effectiveness. The application of MED at 1/2 MIC slows down bacterial growth by extending the lag phase by 25% to 45% when compared with the MED-free control growth curve [196].

#### 5.1.4. Flavanones

**Figure 6:**
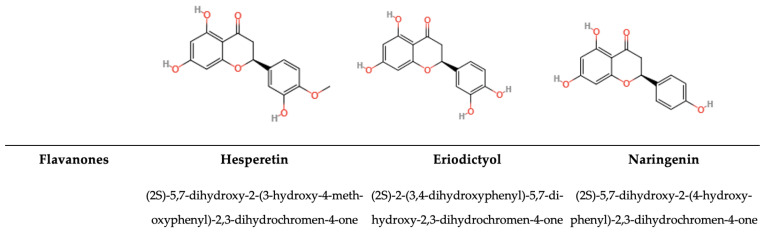
Chemical structure of some representative flavanones and their respective IUPAC names. The molecules were represented using the MolView application (version 2.4).

The configuration of flavanones consists of a flavan nucleus composed of two aromatic rings (A and B) interconnected via a dihydropyrone ring (C) (Figure 6) [198]. The absence of a double bond at the C2–C3 position, the existence of a chiral carbon atom at the C2 site, and the absence of any substitution at the C3 site of the C ring distinguish flavanones from flavones and flavanols [199]. Flavanones are mainly glycosylated, and they occur in cumin, cinnamon, thyme, and oregano in different parts of the plant and in different concentrations [193]. Flavanones are characterized by different substituted derivatives. It is one of the main classes of flavonoids and has 100 glycosylated and approximately 350 aglycone forms, and they occur in different foods, mainly oranges (hesperidin) and lemons (eriodictyol). For example, 200 mL of orange juice contain between 15 and 85 mg narirutin/L and between 200 and 600 mg hesperidin/L [200]. Citrus flavanones have been found to have both antioxidant and anti-inflammatory properties. In particular, various studies have focused their attention on hesperidin and its aglycone form, hesperetin, which play an important role in the prevention of cancer and cardiovascular disease [200]. While flavanones themselves do not exhibit activity against MRSA, compounds such as sophoraflavanone G and exiguaflavanone D are effective due to the hydroxyl group at the C-2′ position of the B ring. Prenylated flavanones, such as macatrichocarpin and the flavanone derivative 4′,7-di-O-methylnaringenin, demonstrate antibacterial properties against *S. aureus*, *B. subtilis, E. coli*, *P. aeruginosa*, *Shigella dysenteriae*, and *Vibrio cholerae* [74].

#### 5.1.5. Flavan-3-ols

**Figure 7:**
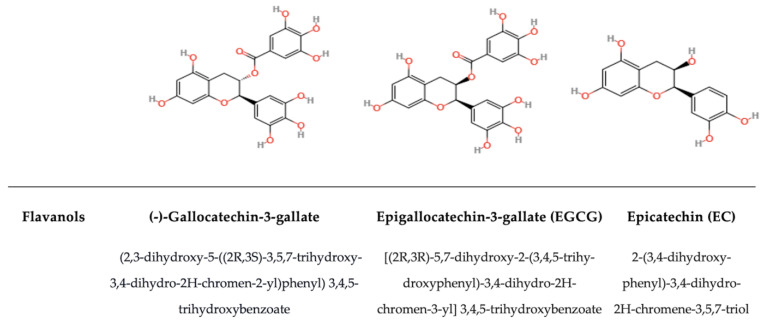
Chemical structure of some representative flavanols and their respective IUPAC names. The molecules were represented using the MolView application (version 2.4).

Flavanols are split into two main groups, depending on their structure: monomeric forms (catechins) and polymeric forms (proanthocyanidins, also called condensed tannins) (Figure 7) [139]. The main compounds of the first group are catechins (Cs), epicatechin (EC), epigallocatechin (EGC), epicatechin gallates (ECGs), and epigallocatechin gallate (EGCG). The main compounds of the proanthocyanidin group are theaflavins (TFs) and thearubigins (TRs). TFs and TRs are transformed during the fermentation of tea leaves and induce the typical colour and flavour of black tea, which contains over 75% of catechins that are converted into complex polymers [201]. Also, EC can polymerize into condensed tannins [202]. These two groups are strictly related and dependent on abiotic conditions. For example, EC in tea exhibits notable stability under heat exposure, provided that the pH remains acidic; at a pH level of 5, only 15% of these compounds are degraded after 7 h in boiling water [141]. This ability to preserve the EC hydroxyl groups’ acidity is crucial for all the relevant functions of interest [203]. The most famous and studied catechin is EGCG, which has many beneficial properties for human health, such as anticancer, anti-obesity, antidiabetic, cardiovascular-protective, anti-infectious, hepatoprotective, and neuroprotective effects. In general, catechins are frequently associated with antibacterial properties through their interactions with the bacterial cell membrane. Unlike flavonoids, catechins have been found to tear bacterial membranes by attaching to the lipid bilayer and inactivating or inhibiting intracellular and extracellular enzymes [204]. Uberos et al. [205] proved that the proteins expressed in cells treated with tea extract were changed. Most of these proteins were enzymes involved in metabolic processes, such as protein biosynthesis, the tricarboxylic acid cycle, DNA metabolism, and fatty-acid biosynthesis. The most prominent functions of polyphenol-interacting proteins are drug metabolism (e.g., cytochrome P450s), oxidation (e.g., aldehyde dehydrogenase), cell cycle regulation (e.g., histone H4 and proteasome), and regulation of metabolism (e.g., protein kinase-like and protein–tyrosine phosphatase) [4]. It has also been observed that catechins kill bacteria by producing reactive oxygen species (ROS), which change membrane permeability and destroy membranes, as in *E. coli* [80,206,207]. Flavanols and other PPs can stimulate the proliferation of *Lactobacillus* spp. and *Bifidobacterium* spp. These molecules can reduce inflammation and enhance metabolism in both healthy and compromised gastrointestinal systems [208]. Furthermore, it has been shown that catechins exhibit a stronger antibacterial effect against Gram-positive than against Gram-negative bacteria.

#### 5.1.6. Anthocyanidins and Anthocyanins

**Figure 8:**
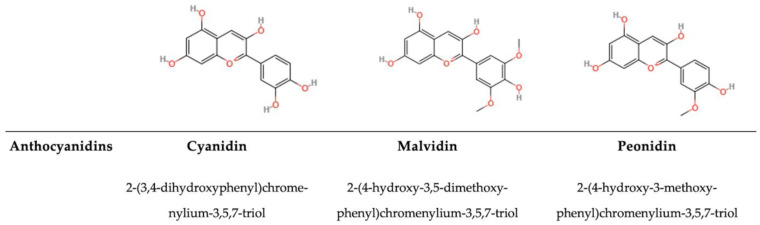
Chemical structure of some representative anthocyanidin and their respective IUPAC names. The molecules were represented using the MolView application (version 2.4).

Their molecular structure consists of a flavylium backbone (2-phenylbenzopyrylium) with various substituents. This core structure includes two aromatic rings (A and B) connected by a three-carbon bridge forming an oxygen-containing heterocycle (ring C) (Figure 8) [141]. Anthocyanidins (ACs) are anthocyanins when glycosylated, and their structure and stability are pH-dependent [209,210]. ACs achieve stability through the formation of complexes with other flavonoids, a process known as co-pigmentation. In human nutrition, anthocyanins are present in red wine, specific types of cereals, and various leafy and root vegetables, such as eggplants, cabbage, beans, onions, and radishes. Catechins are distributed in a variety of foods and herbs, including tea, apples, persimmons, cacaos, grapes, and berries. However, anthocyanins are prevalent in fruits [141]. The structure of ACs contains multiple phenolic hydroxyl groups (polymethoxy and polyhydroxy molecules), which play a vital role in plant communication, defining flower and fruit colours and attracting pollinating agents [210]. Photoprotection through antioxidant enzymes, such as NAD(P)H oxidase, which is also present in animals, is stimulated by anthocyanins, which can induce the activation of different transcription factors that control the expression of antioxidant genes (i.e., the Nrf2-signaling pathway) [186]. Furthermore, anthocyanins and catechins have another positive interaction with the human–gut microbiome, having the potential to boost the population of probiotics such as *Akkermansia* and *Bifidobacterium*, as well as to enhance the proportion of robust bacteria related to *Bacteroides* and intestinal immunity [211,212]. Following the intake of fruits and vegetables, a residue of ACs, flavylium cation, which is another strong antioxidant, remains solely in the stomach, whereas other forms are found in the lower gastrointestinal tract, transforming flavylium carbon into an unstable hemiketal and rapidly becoming a ketone due to keto-enol tautomerism. The resulting α-diketone, which is highly reactive, is rapidly decomposed by the intestinal microbiome into phenolic acid (another strong antioxidant and anti-inflammation substance) [212]. Through this process, cells maintain equilibrium in their vacuoles. In fact, different cellular conditions, such as pH and redox conditions, can modulate the efficiency of ACs. These compounds are thermolabile. Pagliarulo et al. [213] have demonstrated that a crude preparation of anthocyanins is more effective as an antimicrobial than anthocyanins extracted through heat. This is due to the losses of hydroxyl groups, and different extraction methodologies lead to different degrees of antimicrobial effectiveness. Yoon et al. [214] have demonstrated that ACs are more efficient on Gram-negative than on Gram-positive bacteria. Anthocyanins extracted from black beans and the assegai tree inhibit Gram-negative bacteria *(E. coli, P. aeruginosa, C. jejuni, H. pylori,* and *S. enterica)* and Gram-positive bacteria *(L. monocytogenes, S. aureus, B. subtilis, E. faecalis,* and *C. perfringens)* with different target sites, depending on the molecular structure of the ACs studied [215].

#### 5.1.7. Chalcones

**Figure 9:**
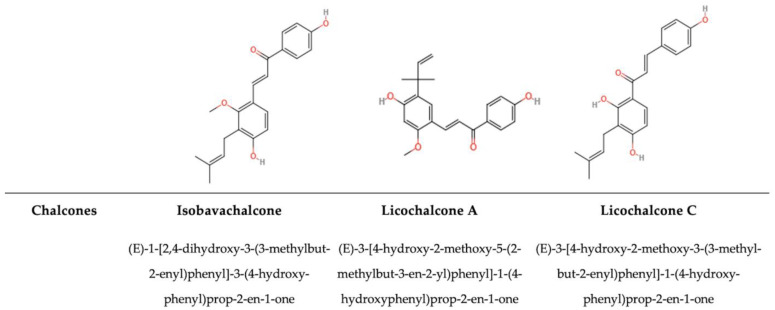
Chemical structure of some representative chalcones and their respective IUPAC name. The molecules were represented using the MolView application (version 2.4).

Chalcones (CHs) are intermediates in the biosynthetic pathway for flavonoids. CHs are a class of natural phenolic compounds characterized by an open-chain structure consisting of two aromatic rings linked by a three-carbon α,β-unsaturated carbonyl system (Figure 9) [216]. This structure provides CHs their distinct yellow colour and contributes to their diverse biological activities, such as antiviral, antimicrobial, antioxidant, and anti-inflammatory properties [217]. CHs can interact with microbes, including bacteria in the gut microbiome [218]. Upon ingestion, they are metabolized by the gut microbiota into bioactive compounds with enhanced biological activities [218]. Tran et al. [219] found that compounds with a hydroxyl group at the C2 or C4 positions in the B ring were involved in the antibacterial activity against MSSA and MRSA, while the hydroxyl group at the C20 position in the A ring was not necessary for activity against these bacterial strains. It was also noticed that the replacement of the aromatic B ring with a heterocyclic ring containing nitrogen, oxygen, or sulphur atoms does not significantly increase the antibacterial activity [220]. Furthermore, chalcones can also interact with different fungi, disrupting the cell membrane or interfering with essential cellular processes (Table 1) [221,222,223,224].

### 5.2. Non-Flavonoids

Non-flavonoid compounds include phenolic acids, stilbenes, and lignans. The main class in this group is represented by phenolic acids, predominantly benzoic acid and cinnamic acid derivatives. The main difference between non-flavonoids and flavonoids is the chemical structure without the typical (C6–C3–C6) [1,74].

#### 5.2.1. Phenolic Acids

**Figure 10:**
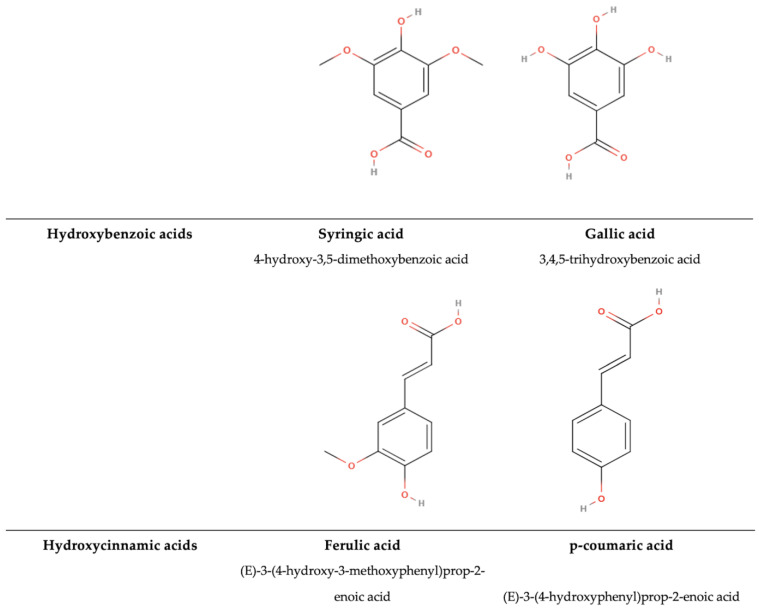
Chemical structure of some representative phenolic acids and their respective IUPAC names. The molecules were represented using the MolView application (version 2.4).

Phenolic acids (PAs), also known as phenol-carboxylic acids, are aromatic acid compounds comprising a phenolic ring and an organic carboxylic acid function (C6–C1 skeleton) (Figure 10) [273]. They encompass hydroxybenzoic acids and hydroxycinnamic acids, derived from non-phenolic molecules of benzoic and cinnamic acid, respectively and Takò et al. [274] have assessed that different PAs can be considered safe for beneficials. PAs derived from plants are compact compounds possessing antimicrobial, antioxidant, anti-inflammatory, and pro-coagulant properties. In addition, they can enhance the physicochemical characteristics of starch, aid in food preservation, serve as natural colourants, and function as prebiotic components [275,276]. Their chemical nature facilitates their potential integration into biomaterial frameworks, offering naturally derived functionalities. Although PAs have been studied before, the relationship between their structure and properties, particularly antioxidant and antimicrobial properties, remains unclear. In general, a trend of slightly decreased antimicrobial efficacy with an increased number of pendant hydroxyl and methoxy groups was observed [273,277]. The antimicrobial effect against *E. coli* is clearly structure dependent (e.g., different substitutions on the benzene ring and the length of the saturated side chain) [275]. Benzoic, phenylacetic, and phenylpropanoid acids fully inhibited *E. coli* at 1000 mg/mL. In contrast, bacteria of the genus *Lactobacillus*, lacking an outer membrane, show different sensitivities based on benzene ring substitutions. Studies on *S. aureus* and *P. aeruginosa* revealed significant differences in susceptibility to 3,4-dihydroxybenzoic acid [229]. Sensitivity to phenolic acids varies among pathogenic microorganisms, such as *S. aureus*, *P. aeruginosa*, and *C. albicans* [276,277]. The lack of an outer membrane in *S. aureus* enables phenolic acids to diffuse through the cell wall, causing intracellular acidification and irreparable damage to the ATPase pump, resulting in cell death. Conversely, the outer membrane of *P. aeruginosa* acts as a barrier against hyper-acidification, making it resistant to phenolic compounds due to efficient toxin-pumping proteins in the (MexE-MexF-OprN) detoxification transport system [278].

#### 5.2.2. Stilbenes

**Figure 11:**
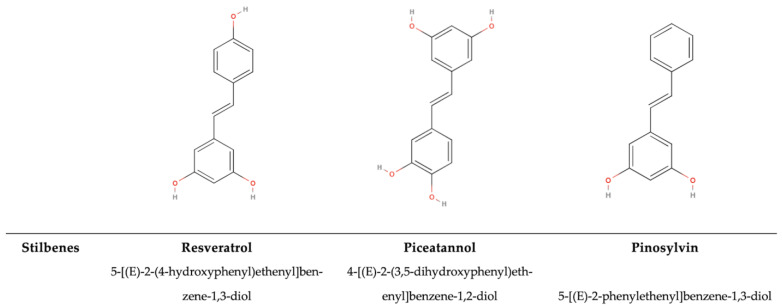
Chemical structure of some representative stilbens and their respective IUPAC names. The molecules were represented using the MolView application (version 2.4).

Stilbenes (C_14_H_12_) are organic compounds that have a compact structure with a central ethylene fraction and one phenyl group (Figure 11) [279]. They are a category of metabolites derived from phenols, and their benefit for human health has been examined in several studies [280]. One of the most well-known stilbenes is resveratrol (3,5,4′-trihydroxystilbene), which is produced by several plants, such as grapes, peanuts, and berries. Resveratrol (RV) has two isomeric forms (*cis* and *trans*). The -*trans* form is the predominant one, and it is the most efficient therapeutic conformation due to the lower steric hindrance of its side chain [6]. However, *Trans*-RV has an inhibitory effect on the growth of bacteria (*Oenococcus oeni, Lactobacillus hilgardii*, *Pediococcus pentosaceus, Acetobacter aceti, Acetobacter oeni*, and *Acetobacter pasterianus*) and yeast *(Dekkera bruxellensis, Hanseniaspora uvarum, Zygosaccharomyces bailii,* and *Zygosaccharomyces rouxii)* [281]. Considering these antimicrobial properties, researchers have recently focused on the use of phenolic extracts as novel antimicrobial agents during the preparation of foods; for example, as a total or partial alternative to traditional treatments with sulphur dioxide (SO_2_) in wine and nitrites/nitrates in foods [282]. Overall, resveratrol showed lower efficacy against Gram-negative bacteria (MIC > 200 mg/mL) than against Gram-positive bacteria (MIC < 200 mg/mL). Indeed, it is more effective against *L*. *monocytogenes* and *L. innocua* than against *H. pylori* [283]. This suggests that resveratrol’s antibacterial action might involve interactions with cytoplasmic or periplasmic targets in Gram-negative bacteria [284]. Furthermore, tannins, oligophenol polymers known for their strong protein adsorption capabilities, are found in vacuoles and waxes, and they can, for example, disrupt the digestive enzymes of pathogens and herbivores [285].

#### 5.2.3. Lignans

**Figure 12:**
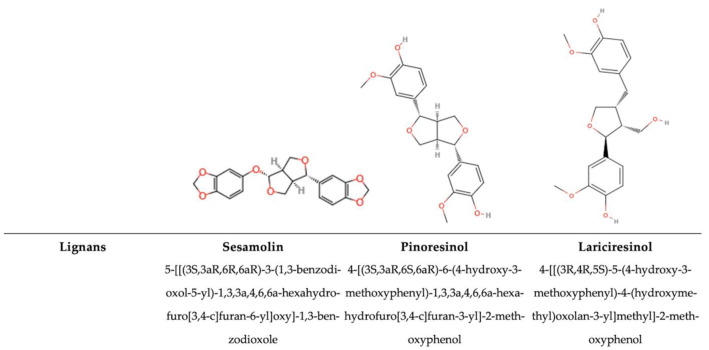
Chemical structure of some representative lignans and their respective IUPAC names. The molecules were represented using the MolView application (version 2.4).

Lignans (LIs) are a group of polyphenolic compounds found in plants and are characterized by their structural backbone consisting of two phenylpropane units linked by a central carbon–carbon bond (Figure 12) [286]. They are categorized into two main classes: dibenzylbutyrolactone derivatives and furofuran derivatives [287]. The most common compounds are diphenolic compounds, widely distributed in different foods, of which the most important ones are grains, vegetables, berries seeds, nuts, tea, and coffee [288]. The most abundant dietary source of lignans is flaxseed, which contains secoisolariciresinol (up to 3.7 g/kg dry weight) and small amounts of matairesinol (these are two of the main eight subclasses of LIs). Interestingly, the concentration of secoisolariciresinol in flaxseed is more than 1000 times higher than in other food sources [289]. The precise physiological function of plant lignans remains unidentified. However, in vitro research has demonstrated that in the human body, lignans are converted into enterodiol and enterolactone by the gut microbiota, and they may exhibit anti-inflammatory properties and other cellular activities that modulate the gut microbiota [287]. Lignans exhibit notable antioxidant and antimicrobial activities. Sesamol, a prominent lignan, has a high radical-scavenging capacity, with an IC50 value of 5.44 μg/mL, comparable to that of butylated hydroxytoluene (BHT). This potent antioxidant effect is largely attributed to the presence of a free-hydroxyl group, enabling sesamol to neutralize free radicals and inhibit lipid peroxidation effectively. Additionally, sesamol has been shown to possess significant antimicrobial properties, with a minimum inhibitory concentration (MIC) of 2 mg/mL against different foodborne pathogens, such as *B. cereus* and *S. aureus*. However, other lignans, such as sesamin and sesamolin, exhibit relatively lower antimicrobial activity [290]. In conclusion, further investigation is needed to identify the proper applications of lignans and to exploit their properties in sustainable food production and preservation.

## 6. Description and Classification of Unclassified Polyphenols: Curcuminoids, Tannins, and Tyrosol Derivatives

**Figure 13:**
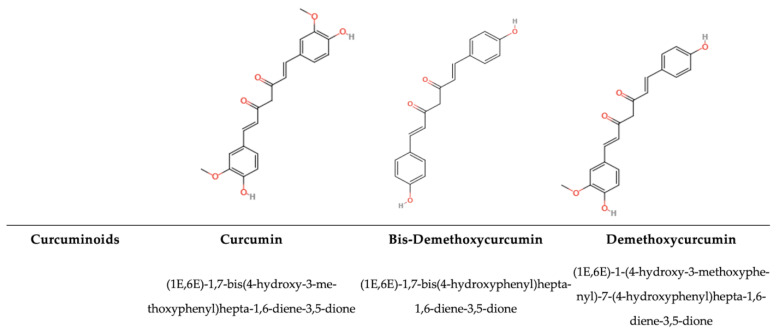
Chemical structure of some representative curcuminoids and their respective IUPAC names. The molecules were represented using the MolView application (version 2.4).

Curcuminoids (CMs) are a vast group of different molecules, with curcumin being the most well-known one. Chemically, curcumin is diferuloylmethane mixed with its two derivatives, dimethoxy-curcumin and bis-dimethoxy-curcumin, with the chemical formulae C_21_H_20_O_6_, C_20_H_18_O_5_, and C_19_H_16_O_4_, respectively (Figure 13) [291]. Curcumin is a highly reactive molecule able to interact with many molecular targets, such as transcription enzymes, protein kinases, receptors, cell cycle regulators, and adhesion molecules [292]. Curcuminoids are strictly confined to plant species, and the relative abundance of each component can have a direct impact on Gram-positive bacteria (*B. subtilis*, *B. cereus*, *E*. *faecalis*, *L. monocytogenes*, and *S. aureus*) and Gram-negative bacteria (*E. coli, P. aeruginosa*, and *Salmonella tiphy*) [293].

**Figure 14:**
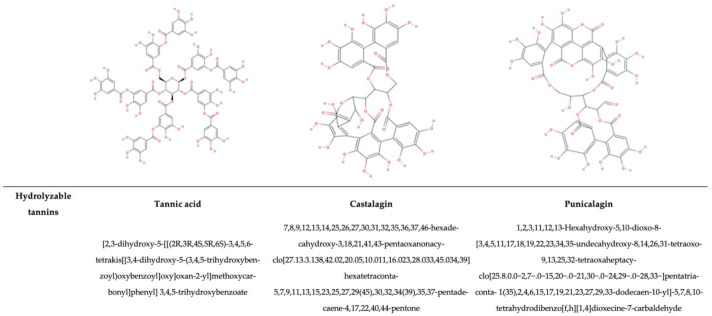
Chemical structure of some representative hydrolysable tannins and their respective IUPAC names. The molecules were represented using the MolView application (version 2.4).

Tannins (TAs) are a diverse group within the phenolic compounds (phenolic hydroxyl groups attached to aromatic rings) and are widely distributed in nature. They are synthesized in the fruits, wood, and bark of trees, as well as in many types of wild herbs and plants. They have molecular weights between 500 and 20,000 Da and very different chemical structures [294,295]. TAs can be grouped into gallotannins, ellagitannins, condensed tannins (CTs), complex tannins (CoTs), and phlorotannins (PTs), which is a group of tannins found in different algal species belonging to the Phaeophyceae class (Figure 14) [295,296]. Polyflavonoid tannins belong to this last group and are rarely hydrolysable; they are also called proanthocyanidins. Proanthocyanidins and CTs are the most common (over 90%) commercially produced tannins worldwide [297]. Tannic acid can reduce lipid oxidation induced by ferrous ions in a plant-based emulsion of flaxseed oil droplets [298,299]. This effect is related to tannins’ chemical structure, which includes phenolic rings capable of binding a wide range of molecules. They act as electron scavengers, trapping ions, and radicals, showing metal-binding properties [298]. They also present many hydroxyl groups, resulting in hydrophilic properties, solubility in aqueous solvents, and the ability to form complexes with proteins, carbohydrates, nucleic acids, and alkaloids [300]. Tannins were applied to ground chicken breast meat to determine their ability to reduce 14 of 22 lipids, influence protein oxidation, maintain colour, and prevent rancid volatiles. The addition of 5–10 ppm of tannic acid to ground chicken breast meat improved the quality parameters of both cooked and raw meat, showing low oxidation markers, low off-odour volatiles, and high colour parameters [140]. Extracts of tannin-rich plants, such as quebracho and conifers, have also been tested as natural food preservatives to improve the shelf life of different products, with promising results. On the other hand, higher concentrations reduced the meat’s softness, tenderness, and juiciness. However, the multiple interactions of tannins, driven by their hydrophilic and nucleophilic groups, enhance their technological potential [301,302]. Because of the organoleptic importance of foods, different tests have been performed on different tannin sources. One study, in particular, analysed conifer tannins as antioxidants in a liposome model and meat snacks. The tested tannins demonstrated strong activity in preventing lipid oxidation without causing organoleptic interference in meat snacks [303]. Altogether, tannins, like other PPs, could be added to food matrices as alternative antioxidant and antibacterial compounds. For example, ellagitannins inhibit the growth of *E. coli, C. perfringens, S. aureus,* and *B. cereus* [303,304]. Furthermore, Wei [304] reported that it is possible to prevent the development of bacterial resistance to beta-lactam antibiotics, such as penicillin and cephalosporin, through the inhibition of beta-lactamase synthesis due to interaction with condensed tannins.

**Figure 15:**
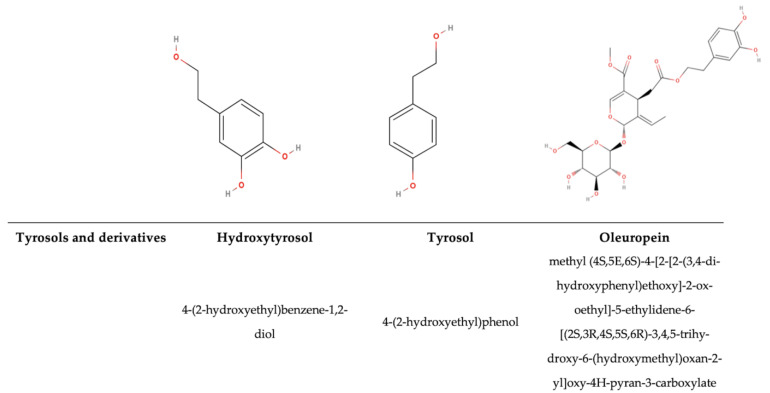
Chemical structure of some representative tyrosol and derivatives, and their respective IUPAC names. The molecules were represented using the MolView application (version 2.4).

Tyrosol (TY) is a phenolic compound derived from pathways such as the pentose phosphate, shikimate, and phenylpropanoid pathways and is a significant secondary metabolite in plants, playing vital roles in their physiology and morphology [305,306]. Olive leaves contain oleuropein and its derivatives, such as hydroxytyrosol (HT) and tyrosol (Figure 15), and a wide array of phenolic derivatives, including simple phenols, flavonoids (such as flavones, flavanones, flavonols, and flavan-3-ols), and secoiridoids [307]. It is reported that olive leaves have antioxidant, hypoglycaemic, antihypertensive, antimicrobial, antitumoral, antiatherogenic, cardioprotective, and antiviral properties [308]. Olive leaf extracts selectively reduce the levels of *Campylobacter jejuni* and *H. pylori*, regulating the composition of the gastric flora and gut microbiome [309]. Different tests have shown that TY alone is more antioxidant than antimicrobial, having a good result against *P. aeruginosa* but no effect on *E. coli*, *L. monocytogenes*, and *S. aureus* [218]. Furthermore, it has been demonstrated that tyrosol can interact with quorum-sensing activities in multiple microorganisms, such as *Candida albicans*, interacting with virulence factors, biofilm formation, and drug efflux under various conditions [310,311]. Although more researchers have recently paid attention to the antibacterial activity of olive leaves, the underlying mechanisms are not fully understood [311,312]. The molecular interaction between pathogen bacteria and HY is clearer. It has been shown that HY acetate interacts with DNA, with different binding activities, and the phospholipid bilayer, leading to its disruption [218]. It has been reported that the antimicrobial activity of olive leaves is related to their terpene content; terpenes act by disrupting cell membranes [313,314]. Furthermore, technologically speaking, HT and T can be used as a stabilizer and an antioxidant in foods or industrial preparations. Possibly due to their hydrophilic nature and problems associated with their extraction from aqueous solutions and solubilization in lipid-based systems, TY and HTY are generally well absorbed by cells, thus preventing different oxidative stress conditions [315] as assessed by the health claims by EFSA about hydroxytyrosol. Due to its confirmed positive interactions already cited and a risk assessment for HY, hydroxytyrosol has been declared a food ingredient, confirming its healthy and safe utilization (Table 2). 

## 7. Conclusions

The exploration of the antimicrobial potential of PPs has unveiled their multifaceted interactions with microbial structures and functions. PPs exert their effects through diverse mechanisms, including interactions with cell membranes, DNA and RNA, quorum-sensing systems, and cellular energetics, such as membrane depolarization and ATP production. Additionally, the complex interplay between PPs and metal ions highlights their capability to interfere with essential microbial processes. These insights underline the dual structural and biological significance of PPs, which play a critical role in inhibiting microbial growth and modulating their physiological responses. Furthermore, the distinction between extractable and non-extractable PPs expands our understanding of their bioavailability and potential applications. NEEPs, often overlooked, present promising opportunities in advancing health and environmental sustainability due to their persistence, distribution in crops, and gradual release of bioactive compounds. This narrative review gains significance in the context of a growing societal shift towards healthier lifestyles and environmentally friendly practices. The increasing consumer demand for “green labels” and organic products drives the development of innovative, eco-friendly, and health-conscious solutions. Advanced detection technologies and the integration of by-products into human health management further support these goals. By reviewing the state-of-the-art modes of action and cellular interactions of polyphenolic extracts, this research contributes to addressing global challenges, such as antibiotic resistance, the rise in foodborne outbreaks, and the reduction of environmental impacts. Due to the opportunities provided by our ecosystem, it is essential to deepen our understanding of PPs, both extractable and non-extractable, as well as functional foods. These efforts align with the Agenda 2030 and the Sustainable Development Goals [350], paving the way for a future in which antimicrobial strategies are more effective and sustainable in our ecosystem. In conclusion, continued research in this field is imperative to fully harness the potential of PPs as a cornerstone of innovative antimicrobial approaches.

## Figures and Tables

**Table 1:** Flavonoid compounds used as antimicrobials and antioxidants in food are categorized by sub-classes, compound name, source of the studied PPs, bacterial interactions, sites of interaction, MICs, and references.

Reference	MIC	Antimicrobial	Microbial Target	Source	Compounds	Subclass Name
		Activity				
[74,203,215,225,226,227,228,229,230]	*B. cereus*: 0.43 ± 0.14 nmol/well	Interaction with bacterial membrane, inhibition of different metabolic and replicative enzymes, induction of ROS production	*B. cereus, E. coli*, and *S. aureus*	Tea, cocoa, peach, raspberry, apple, red grape	(-)-Gallocatechin-3-gallate (GCg)	Flavanols
	*S. aureus*: 133 µg/mL	ROS production, efflux pump inhibitor, membrane disrupting, DNA-replication inhibitor, leakage citoplasmatic content, interaction with FabG, FabI in the bacterial FAS-II	*V. cholerae, Streptococcus mutans,*	Tea leaf, apple, pecan nut, plums	Epigallocatechin-3-gallate (EGCg)	
	*B. cereus*: 267 µg/mL		*C. jejuni, C. perfringens, E. coli*			
	*B. subtilis*: 533 µg/mL					
	*C. perfringens*: 50–400 µg/mL *L. monocytogenes*: 400 µg/mL *V. parahaemolyticus*: 83 µg/mL *E. coli*: 533 µg/mL					
	*Y. enterocolitica*: 333 µg/mL *P. aeruginosa*: 400 µg/mL					
	*S. aureus*: 133 µg/mL	Efflux pump interaction and inhibition, inhibition of ATPase	*L. monocytogenes, S. mutans, Salmomella *spp.*, C. jejuni,*	Blackberry	Epicatechin gallate (ECg)	
	*B. cereus* 200 µg/mL		*V. parahaemolyticus*	Raspberry, apple, grape		
	*B. subtilis* 533 µg/mL					
	*C. perfringens*: 100 µg/mL *L. monocytogenes*: 400 µg/mL					
	*V. parahaemolyticus*: 67 µg/mL *E. coli*: 533 µg/mL					
	*Y. enterocolitica*: 400 µg/mL *P. aeruginosa*: 667 µg/mL					
[68,74,231,232,233,234,235,236,237,238]	*E. coli*: 500 µg/mL	Inhibition of FAS II, inhibition of topoisomerase IV enzyme, depolarization cellular membrane, efflux pump inhibitors (NorA) S. aureus, pro-oxidant	*P. aeruginosa, B. subtilis, S. aureus, E. coli, S. enterica*	Cranberry, *Camellia sinensis*, parsley, apple, blackberry, pomegranate, onion, garlic, broccoli, kale, eggplant, spinach, bean	Kaempferol	Flavonols
	*P. aeruginosa*: 8 µg/mL					
	*S. aureus*: 50 µg/mL	Inhibition of nucleic acid synthesis, membrane interactions, inhibition of topoisomerase IV enzyme	*S. aureus, K. pneumoniae*	Galangal root, propolis, *Helichrysum* sp.	Galangin	
			*B. cereus, B. subtilis, E. coli*			
	*B. subtilis*: 250 µg/mL	Inhibition of DNA helicase and gyrase, ATPase activity, pro-oxidant	*S. aureus, B. subtilis, E. coli,*	*Myrica* sp., tea leaf, blackberry, cranberry	Myricetin-3-O-rhamnoside	
	*E. coli*: 250 µg/mL		*K. pneumoniae*			
	*K. pneumoniae*: 250 µg/mL *S. aureus*: 250 µg/mL		*L. monocytogenes, S. mutans*			
	*C. albicans*: 130 µg/mL		*Salmomella *spp.*,*			
	*C. jejuni, V. parahaemolyticus, C. albicans*					
	
	*B. subtilis*: >250 µg/mL	Alteration sortase activity in S. aureus, pro-oxidant, antimicrobial, antifungal	*S. aureus, B. subtilis, E. coli*	Apple, hawthorn, caper, onion, green tea	Quercetin-3-O-rhamnoside	
	*E. coli*: >250 µg/mL *K. pneumoniae*: >250 µg/mL *S. aureus*: >250 µg/mL		*K. pneumoniae, C. albicans*			
	*C. albicans*: 20 µg/mL					
	*B. subtilis*: 30 µg/mL	Reduction of adherence during biofilm formation, prevent ATP hydrolysis, changes membrane permeability, induction membrane damage	*S. aureus, B. subtilis, E. coli*	Onion, berries, broccoli, tea leaf, apple, grape	Quercetin	
	*E. coli*: 250 µg/mL		*K. pneumoniae, P aeruginosa*			
	*K. pneumoniae*: 250 µg/mL *C. albicans*: 20 µg/mL *Clostridium* spp.: <15 µg/mL *Salmonella* spp.: <20 µg/mL		*Mycobacterium tuberculosis*			
			*C. albicans*			
[77,215,239,240,241,242,243,244,245,246,247,248]	*H. pylori*: 100 μM	Lag phase duration increased, interaction with FAS-II, inhibitor of *S. aureus* exotoxin*,* efflux pump inhibitor	*L. monocytogenes, B. cereus*	Soy, red clover, alfalfa, kidney and common bean, lupin, peanut	Genistein	Isoflavones
	* B. brevis*: 17.5 µg/mL * B. subtilis*: >128 µg/mL * E. coli*: >128 µg/mL		*S. aureus*			
	*P. fluorescens*: >50 µg/mL *S. aureus*: >128 µg/mL *S. enterica*: >128 µg/mL		*V. parahaemolyticus*			
			*Salmonella* Typhimurium,			
			*H. pylori*			
	*B. brevis*: 17.5 µg/mL *S. typhimurium*: 64 µg/mL *B. subtilis*: 64 µg/mL *E. coli*: 64 µg/mL	Membrane interaction and disruption, inhibition of DNA gyrase, inhibition of biofilm formation, efflux pump inhibitor	*E. coli, S. aureus, S. epidermidis*	Soy, mango, peach, prune, strawberry	Daidzein	
	*P. aeruginosa*: 64 µg/mL *P. vulgaris*: 64 µg/mL		*Listeria* spp., *P. aeruginosa*			
	* S. aureus*: 64 µg/mL		*H. pylori*			
	*K. pneumoniae*: 125 µg/mL	Induction of oxidative damage, inhibition of biofilm formation	*S. aureus, K. penumoniae, B. cereus, B. subtilis,* *E. Coli*	Soy, fava bean, chickpea	Glycitein	
[68,175,215,249,250,251]	*E. faecalis, E. coli,*	Destabilization of membrane structure, cytoplasmic leakage, inhibition of FAS-II, inhibition of gyrase	*S. aureus, B. cereus, K. pneumoniae, P. vulgaris, S. typhi, E. coli*	Parsley, celery, chamomile, orange	Apigenin	Flavones
	*P. aeruginosa*: 500–1000 µg/mL		*H. pylori*			
	*P. aeruginosa*: 100–200 µg/mL	Inhibition of FAS II, inhibition of ATPase, inhibition of DNA and RNA polymerase, inhibition of NorA pump and efflux pump	*Enterecoccus* spp., *M. tuberculosis, E. coli, S. aureus, MRSA*	Thyme, baikal skullcap	Baicalein	
			*P. aeruginosa, C. albicans*			
	*S. aureus*: 64–128 µg/mL	Inhibition of FAS II and DNA topoisomerase, interaction with liposomal membrane	*S. aureus, MRSA, Enterococcus* spp., *E. coli, M. tuberculosis,*	Celery, green pepper, thyme, carrot, chamomile	Luteolin	
	*B. cereus, B. subtilis,*		*L. monocytogenes,* *B. subtilis*			
	*L. monocytogenes,* *P. aeruginosa:* 19–156 µg/mL					
[215,251,252,253,254,255,256,257]	*C. violaceum*: 150 μg/mL *K. pneumoniae*: 250 μg/mL *S. aureus*: 270 μg/mL *V. parahaemolyticus*: 130 μg/mL	Inhibition of QS, pro-oxidant, metal complexation	*P. aeruginosa, E. coli, S. aureus*	Strawberry, tea, red wine, blueberry, cranberry, cherry	Cyanidin	Anthocyanins
			*E. faecalis, S. aureus,*			
			*Salmonella enteritidis*			
			*K. pneumonia, S. mutans*			
	*S. aureus*: 270 μg/mL *V. parahaemolyticus*: 130 μg/mL	Disruption membranes, ROS generation, inhibition of biofilm adherence, growth, and formation	*K. penumonia, S. aureus*	Strawberry, grape, blackberry, cranberry	Malvidin	
	*S. aureus*: 270 μg/mL *V. parahaemolyticus*: 130 μg/mL	Disruption of cell membrane and leakage of intracellular contents, ROS production, interaction with QS	*E. coli, Salmonella* spp., *L. monocytogenes, S. aureus*	Blackberry, prune, cranberry, grape	Peonidin	
[175,258,259,260,261,262,263,264,265,266]	*S. aureus*: 4000 µg/mL *Candida* spp.: 165 µg/mL	Interaction with FAS-II	*B. cereus, P. aeruginosa, S. aureus, E. Coli*	Orange, mandarin, lime, lemon, grape fruit	Hesperetin	Flavanones
	*P. aeruginosa*: 0.012–0.22 mmol/mL *K. pneumoniae*: 0.012–0.22 mmol/mL *S. aureus*: 512–1000 µg/mL *E. coli*: 250 µg/mL	Interaction with FAS-II, depolarization of the cellular membrane, inhibition of DNA, RNA, and protein synthesis, interaction with *E. coli* FabI enzyme	*S. aureus, L. monocytogenes*	Lime, tea plant, gum plant	Eriodictyol	
	*S. enterica*: 800 µg/mL		*Salmonella* Typhimurium			
	*B. subtilis*: 250 µg/mL		*P. aeruginosa, S. enterica*			
	*L. innocua*: 800 µg/mL		*E. coli*			
	*S. aureus*: 512–1000 µg/mL *E. coli*: 800 µg/mL	Interaction with FAS-II, antibiofilm, downregulation of QS genes	*Salmonella* Typhimurium	Grapefruit, orange, tomatoes, cocoa, bergamot	Naringenin	
	*S. enterica*: 1000 µg/mL *B. subtilis*: 1000 µg/mL *L. innocua*: >1000 µg/mL		*L. monocytogenes, S. aureus, MRSA, L. rhamnosus, B. subtilis, Micrococcus luteus, E. coli,*			
			*P. aeruginosa, S. enterica*			
[267,268,269,270,271,272]	*E. coli*: 39.1 µg/mL	Inhibition of oxygen consumption and biofilm formation, reduce ATP synthesis, inhibition of nuclease activity, membrane disruption and intracellular leakage, oxidative stress induction	*S. mutans, S. aureus, MRSA*	Fabaceae, Moraceae	Isobavachalcone	Chalcones
	*K. pneumoniae*: 39.1 µg/mL *P. aeruginosa*: 39.1 µg/mL *S. dysenteriae*: >39.1 µg/mL *S. faecalis*: 0.3 µg/mL		*E. faecalis, B. cereus*			
	*S. aureus*: 0.3 µg/mL *B. cereus*: 0.6 µg/mL		*M. tubercolosis, E. coli*			
			*P. aeruginosa*			
	*E. coli*: >128 µg/mL *MRSA*: 1–8 µg/mL	Inhibition of NADH-cytochrome c reductase activity, antibiofilm, reducing pathogenicity	*S. aureus, E. coli, S. epidermidis*	Soy and legumes, soy products	Licochalcone A	
			*L. monocytogenes,* *B. cereus*			
			*Cronobacter sakazaki*			
			*Shigella sonnei, Legionella* spp., *Mycobacteria* spp., *C. albicans*			
			*A. niger*			
	*E. coli*: >250 µg/mL *MRSA*: 1*–*16 µg/mL	NADH oxidase, NADH-cyt.C reductase	*B. subtilis, S. aureus, MRSA*	Soy and legumes, soy products	Licochalcone C	
			*S. cerevisae, E. coli,* *P. aeruginosa*			

**Table 2:** Non-flavonoid compounds used as antimicrobials and antioxidants in food, categorized by sub-classes, compound name, source of the studied PPs, bacterial interactions, sites of interaction, MICs, and references.

References	MIC	Antimicrobial	Microbial Target	Source	Compounds	Subclass Name
		Activity				
[74,280,283,316,317,318,319,320,321,322,323,324,325]	*S. aureus*: 512–2133 μg/mL	Interaction with signaling pathways and QS, interaction with FASII, reduction of toxin production, inhibition of biofilm formation, reduction of motility	*S. aureus, B. cereus, E. coli, B. subtilis,*	Grape, wine, peanuts, cocoa, blueberry	Resveratrol	Stilbenes
	*B. cereus*: 2667 μg/mL		*C. perfringens*			
	*B. subtilis*: 2667 μg/mL *L. monocytogenes:* 50–2133 μg/mL		*L. monocytogenes*			
	*E. coli*: 3200 μg/mL					
	*P. aeruginosa*: 2133 μg/mL					
	*V. parahaemolyticus:* 333 μg/mL *Y. enterolitica*: 2133 μg/mL					
	*P. aeruginosa*: 128 μg/mL *S. aureus*: >512 μg/mL	Inhibition signaling pathways, pro-oxidant, inhibit bacterial ATP synthesis, DNA cleavage, antibiofilm	*C. perfringens*	White tea, red wine, grape, passion fruit	Piceatannol	
			*S. aureus, MRSA,*			
			*S. mutans, S. suis,*			
			*S. typhi, P. aeruginosa, S. dysenteriae, E. coli,*			
			*K. pneumoniae*			
	C. jejuni: 25 μg/mL *Campylobacter coli*: 50 μg/mL *S. aureus*: 25 μg/mL	Membrane depolarization and disruption, pro-oxidant, interaction with cellular respiration	*E. coli, S. aureus,*	*Pinus sylvestris, Picea abies, Pinus* spp.	Pinosylvin	
			*L. monocytogenes, P. fluorescens,*			
			*L. plantarum,*			
			*C. albicans,*			
			*C. coli*			
			*C. jejuni*			
[74,323,326,327]	*B. subtilis*: <5 μg/mL *Bacillus* spp.: <5 μg/mL *B. cereus*: NA	Membrane disruption, metabolism interaction ATP synthase, anti-QS (biofilm)	*E. coli. S. aureus,*	Grape, blueberry, olive, onion, tomato, oat, coffee	Syringic acid	Hydroxybenzoic
			*L. monocytogenes,*			acids
			*B. subtilis,*			
			*P. aeruginosa,*			
			*C. albicans*			
	*B. cereus*: 1067 μg/mL	Disruption of cytoplasmatic membrane, intracellular potassium release, intracellular leakage, inhibition motility	*E. coli, S. aureus,*	Bearberry, gallnut, tea leaf, grape, black currant	Gallic acid	
	*B. subtilis*: 1600 μg/mL		*L. monocytogenes,*			
	*C. perfringens*: 83 μg/mL		*B. subtilis,*			
	*S. aureus*: 533 μg/mL		*P. fluorescens*			
	*L. monocytogenes*: 1600 μg/mL *P. aeruginosa*: 533 μg/mL *V. parahaemolyticus*: 50 μg/mL *Y. enterolitica*: 533 μg/mL		*P. aeruginosa*			
	*E. coli*: 600 μg/mL					
	*P. aeruginosa*: 100 mg/mL *E. coli*: 100 mg/mL *S. aureus*: 1100 g/mL *L. monocytogenes*: 1250 mg/mL	Interference with membrane potential, interaction with cytoplasm pH, membrane disruption	*S. aureus, B. cereus*		Ferulic acid	Hydroxycinnamic acids
			*L. monocytogenes*	Strawberry, blackberry, tea, onion, wine, coffee, apple, tomato, spinach, extra virgin olive oil		
			*E. coli*			
			*Shigella dysenteriae, Salmonella*			
			Typhimirium			
	*B. cereus*: 24 μg/mL *S. aureus*: 24 μg/mL *L. monocytogenes*: 24 μg/mL *E. coli*: 49–80 μg/mL *S. dysenteriae*: 10 μg/mL *Salmonella* Typhimurium: 20–49 μg/mL	Inhibition of DNA and RNA synthesis, membrane cell interaction and leakage, inhibition of RecA protein	*S. dysenteriae, E. coli, S. pneumoniae, S. aureus, B. subtilis,*	Peanut, basil, garlic, tomato, legume	*p*-Coumaric acid	
			*Salmonella*			
			Typhimurium			
[328,329,330,331]	*Mucor circinelloides*: 1.5 mM	Modification of cell wall composition, inhibition of different protein synthesis	*S. aureus, B. cereus*	Soy and legume, soy products	Sesamolin	Lignans
	C. albicans: 12–25 μg/mL Fusarium graminearum: 5 μg/mL	Cellular membrane damage, disruption of cell wall	*P. aeruginosa,*	Strawberry, apple, cranberry, pear, prune	Pinoresinol	
			*B. subtilis,*			
			*S. aureus, E. coli,*			
			*S. enterica*			
	*S. aureus*: 125 μg/mL *E. coli*: 250 μg/mL	Transcriptional repressor,	*L. monocytogenes*	Sesame seeds, tea plant	Lariciresinol	
		antibiofilm	*S. aureus,*			
			*E. coli,*			
			*C. albicans,*			
[74,228,332,333,334,335,336]	*B. cereus*: 533 μg/mL	Inhibition of the NorA efflux pump, binds cell receptors and inhibits virulence protein, interaction with different pathways and metabolic proteins	*E. coli, Klebsiella pneumoniae,*	Oak, sumac, tea plant, chestnut, pomegranate	Tannic acid	Hydrolizable tannins
	*B. subtilis*: 667 μg/mL		*L. monocytogenes*			
	*C. perfringens*: 50 μg/mL *S. aureus*: 400 μg/mL		*S. aureus, A. baumannii*			
	*E. coli*: 1333 μg/mL *L. monocytogenes*: 1600 μg/mL *Y. enterocolitica*: 467 μg/mL *V. parahaemolyticus*: 75 μg/mL *P. aeruginosa*: 400 μg/mL		*Y. enterocolitica*			
			*E. faecalis*			
	*B. cereus*: 467 μg/mL	Interaction with metabolic proteins, disruption of essential cellular pathways	*S. aureus, B. cereus, E. coli, B. subtilis*	Chestnuts, red grapes, red wine, chocolate, tea	Castalagin	
	*B. subtilis*: 600 μg/mL *C. perfringens*: 67 μg/mL *S. aureus*: 267 μg/mL		*C. perfringens*			
	*E. coli*: 533 μg/mL *P. aeruginosa*: 400 μg/mL *V. parahaemolyticus*: 117 μg/mL *Y. enterocolitica*: 400 μg/mL *L. monocytogenes*: 667 μg/mL		*L. monocytogenes*			
	*B. cereus*: 667 μg/mL	Inhibition of biofilm formation, cell membrane damage, toxin inhibition	*S. aureus, B. cereus, E. coli, B. subtilis, C. perfringens, L. monocytogenes*	Pomegranates (peel, seed, and juice)	Punicalagin	
	*B. subtilis*: 467 μg/mL *C. perfringens*: 67 μg/mL					
	*S. aureus*: 600 μg/mL *L. monocytogenes*: 3200 μg/mL *E. coli*: 2133 μg/mL *V. parahaemolyticus*: 83 μg/mL *Y. enterocolitica*: 400 μg/mL *P. aeruginosa*: 533 μg/mL					
[337,338,339,340,341,342,343,344]	*B. cereus*: <15 μg/mL *B. subtilis*: <15 μg/mL *L. monocytogenes*: <20 μg/mL *Clostridium* spp.: <5 μg/mL *E. coli*: <20 μg/mL	Disruption of the biofilm structure, inhibition of the swimming capacity, inhibition of cell division and proliferation (inhibiting the polymerization of FtsZ protofilaments, disturbing GTPase activity)	*S. aureus, MRSA, Streptococcus pyogenes, K. pneumoniae, P. aeruginosa, E. coli, S. mutans, B. subtilis, H. pylori*	*Curcuma* spp.	Curcumin	Curcuminoids
	*Salmonella* spp.: <20 μg/mL					
	*S. aureus 7*.8–15.6 μg/mL	ATPase inhibition and membrane permeability (attacking PBP2a gene), inhibition and downregulation of enterotoxin A (SEA)	*S. aureus, S. mutans*	*Curcuma* spp.	Bis-Demethoxycurcumin	
	*Proteus vulgaris*: 500 μg/mL *V. parahaemolyticus*: 250 μg/mL *E. coli*: 500 μg/mL *S. aureus*: 125 μg/mL *B. cereus*: 125 μg/mL	ATPase inhibition and membrane permeability in bacterial and fungal microorganisms, interference with metabolic protein synthesis, pro-oxidant	*S. aureus, B. cereus, E. coli, MRSA, S. dysenteriae*	*Curcuma* spp.	Demethoxycurcumin	
[313,324,345,346,347,348,349]	*S. aureus*: 7.85–400 μg/mL *S. mutans*: 312 μg/mL	Reduction of intracellular ATP concentration, membrane depolaritazion, metal chelating capability, pro-oxidant	*C. albicans, S. aureus, L. monocytogenes*	Olive oil,	Hydroxytyrosol	Tyrosol and derivatives
	*E. faecalis*: 1250 μg/mL		*K. pneumoniae,*	wine and green tea		
	*E. coli*: ND		*P. aeruginosa*			
	*K. pneumoniae*: ND *P. aeruginosa*: >1000 μg/mL *S. typhi*: 3.94 μg/mL *Vibrio* spp.: 0.97–7.8 μg/mL		*Y. enterocolitica*			
			*Salmonella*			
			Typhimurium,			
			*S. enterica, E. coli*			
	*B. cereus*: 400 μg/mL *Salmonella* Typhimirium: 400 μg/mL *S. aureus* 600 μg/mL *E. coli*: 600 μg/mL	Reduction of intracellular ATP concentration, direct inhibition on metabolism proteins, chelating capability, cell membrane depolarization	*E. coli, P. aeruginosa,* *S. aureus, and L. monocytogenes*	Olive oil,	Tyrosol	
	*H. pylori, C. albicans*: NA *K. pneumoniae*: 600 μg/mL			wine and green tea		
	*S. aureus*: 62.5–3200 μg/mL *S. mutans*: 625 μg/mL	Biofilm interaction, growth rate reduction, bacterial motility reduction, interaction with the outer membrane, promoting its disruption	*S. aureus, MRSA,*	Olive oil,	Oleuropein	
	*E. faecalis*: 1250 μg/mL		*E. coli, S. Enteritidis, L. monocytogenes,*	wine and green tea		
	*P. aeruginosa*: ND		*B. cereus,*			
	*S. typhi*: 125 μg/mL		*K. pneumoniae,*			
	*Vibrio* spp. 62.5–125 μg/mL		*C. jejuni,*			
			*H. pylori, C. albicans*			

## Data Availability

The original contributions presented in this study are included in the article. Further inquiries can be directed to the corresponding author.
